# Design and Synthesis of Hybrid Compounds for Potential Treatment of Bacterial Co-Infections: In Vitro Antibacterial and In Silico Studies

**DOI:** 10.3390/antibiotics14060582

**Published:** 2025-06-06

**Authors:** Vuyolwethu Khwaza, Opeoluwa O. Oyedeji, Eric Morifi, Mutshinyalo Nwamadi, Thierry Youmbi Fonkui, Derek Tantoh Ndinteh, Blessing A. Aderibigbe

**Affiliations:** 1 Department of Chemistry, Alice Campus, University of Fort Hare, Alice 5700, South Africa; ooyedeji@ufh.ac.za; 2 Mass Spectrometry Division, School of Chemistry, University of Witwatersrand, Johannesburg 2000, South Africa; eric.morifi@wits.ac.za; 3 Department of Chemistry, Auckland Park Campus, University of Johannesburg, Johannesburg 2092, South Africa; mnwamadi@uj.ac.za; 4 Department of Biotechnology, Doornfontein Campus, University of Johannesburg, Johannesburg 2028, South Africa; thierryfy@uj.ac.za; 5 Department of Chemical Sciences, Doornfontein Campus, University of Johannesburg, Johannesburg 2028, South Africa; dndinteh@uj.ac.za

**Keywords:** synthesis, hybrid compounds, antibacterial, in silico studies, SwissADME, COVID-19, co-infection

## Abstract

**Background:** The need for innovative therapeutic strategies to enhance patient outcomes has increased due to the rise in bacterial co-infections associated with COVID-19. **Methods:** In this study, ten hybrid compounds were synthesized by combining two known pharmaceutical scaffolds to enhance antibacterial activity and overcome resistance mechanisms. The synthesized compounds were evaluated for their antibacterial activity against five Gram-negative and seven Gram-positive bacterial strains. In silico pharmacokinetic and drug-likeness properties of selected active compounds (**12**–**16**, **19**, **21**, and **23**) were predicted using the SwissADME web tool. **Results:** Compounds **12–16**, **19**, **21**, and **23** demonstrated significant antibacterial activity, with compound **16** (a ciprofloxacin-containing hybrid) exhibiting the most potent effect, showing a minimum inhibitory concentration (MIC) of 7.8125 µg/mL against all tested bacterial strains. The in silico analysis revealed favorable pharmacokinetic profiles, drug-likeness, lipophilicity, and water solubility of most hybrid compounds. **Discussion:** The synthesized hybrid compounds exhibited enhanced antibacterial activity and desirable pharmacokinetic properties, particularly compound **16**. These findings suggest the potential of these molecules in combating bacterial pathogens, especially those implicated in co-infections in COVID-19 infections. **Conclusions:** The study presents promising hybrid antibacterial agents with potential application as adjunct therapies for treating COVID-19-associated bacterial co-infections. Further investigation is needed, which may lead to effective treatments for managing secondary bacterial infections in viral disease contexts.

## 1. Introduction

The COVID-19 pandemic demonstrated the complexity of viral infections, especially with the concurrent rise in bacterial co-infections that complicate clinical outcomes [1]. Bacterial co-infections in COVID-19 patients have been linked to prolonged hospital stays, increased mortality, and an elevated need for intensive care. The risk of bacterial co-infections alongside COVID-19 continues to be a significant concern, although the WHO downgraded the status of COVID-19 in 2023 [2]. These co-infections are often caused by opportunistic bacteria, which frequently lead to respiratory complications, bloodstream infections, and sepsis, especially in immunocompromised individuals or those requiring mechanical ventilation [3]. *Bacillus subtilis* [4] and *Enterococcus faecalis* [5] have been detected in infected individuals. Giacobbe et al. [6] reported that the predominant bacterial co-pathogens in SARS-CoV-2 infections include *Streptococcus pneumoniae*, *Staphylococcus aureus*, and *Klebsiella pneumoniae*. Interestingly, Zhong et al. [7] found that *Staphylococcus epidermidis* was among the most common respiratory microbial taxon in critically ill patients. A study by Basnet et al. on 49 hospitalized COVID-19 patients found that 6.12% were co-infected with uropathogenic *Escherichia coli* (95% confidence interval: 0.59–12.83) [8]. Additionally, Pintado’s research [9] highlighted an increased risk of carbapenemase-producing *Enterobacter cloacae* infections in COVID-19 patients, which often present as severe nosocomial infections with high mortality rates. Koupaei et al. [10] reported a *Klebsiella* spp. co-infection rate of 42.91% in ICU-admitted patients, primarily involving *K. oxytoca* (6.7%), *K. aerogenes* (9.4%), and *K. pneumoniae* (26.81%). Overall, bacterial infections were found in 15.5% of hospitalized COVID-19 patients, with a mortality rate of 32.5% among co-infected cases. The situation is further exacerbated by the widespread use of antibiotics, which has led to increased antimicrobial resistance (AMR), rendering many standard treatments ineffective [11].

Hybrid compounds have emerged as a promising class of therapeutics that combine two or more bioactive pharmaceutical scaffolds into a single molecule, potentially enhancing antibacterial potency while minimizing resistance [12,13]. This method takes advantage of the synergistic effects of the combined pharmacophores, which could result in molecules with reduced side effects and increased biological activity [13,14,15]. These compounds can act on multiple targets within bacterial cells, thus improving efficacy and reducing the likelihood of resistance development [14,16,17]. Moreover, hybrid molecules have been shown to exhibit unique pharmacokinetic and pharmacodynamic profiles [18], offering potential advantages in managing co-infections by broadening antibacterial coverage and reducing the dosage needed for effective treatment.

**Figure 1:**
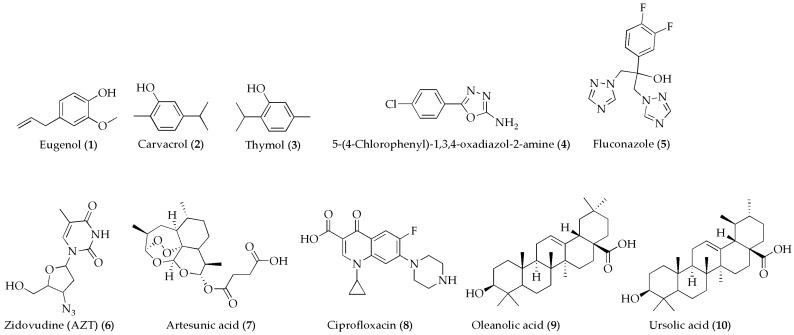
Selected scaffolds: eugenol (**1**), carvacrol (**2**), thymol (**3**), 5-(4-Chlorophenyl)-1,3,4-oxadiazol-2-amine (**4**), fluconazole (**5**), zidovudine (**6**), artesunic acid (**7**), ciprofloxacin (**8**), oleanolic acid (**9**) and ursolic acid (**10**).

This study focuses on developing hybrid compounds as potential therapeutic drugs for bacterial co-infections associated with COVID-19. The antibacterial properties of these hybrid molecules were tested against specific bacterial pathogens reported in the literature as being related to bacterial co-infections in COVID-19 patients. By strategically combining pharmacophores (in Figure 1) with known pharmacological properties, we hypothesize that these hybrid molecules will exhibit enhanced antibacterial activity, particularly against multidrug-resistant strains commonly found in co-infected COVID-19 patients. We also conducted an in silico evaluation of the synthesized hybrid compounds to predict their pharmacokinetic profiles, drug-likeness, physicochemical properties, lipophilicity, water solubility, and medicinal chemistry to gain insights into their pharmacokinetic behaviors using SwissADME. The findings from this research could provide a basis for new therapeutic options that address the bacterial challenges posed by COVID-19.

In this study, compounds **1**–**10**, as shown in Figure 1, were explored for hybridization due to their potent therapeutic properties, such as antibacterial, antifungal, and antiviral properties. Numerous research studies have observed essential oils extracted from various aromatic and medicinal plants to have potent antibacterial activity against resistant microbial strains [19,20,21,22]. This antibacterial effect is attributed to phenolic compounds, including thymol (**3**), carvacrol (**2**), and eugenol (**1**), which are recognized as key constituents of most essential oils [23]. Among the antifungal drugs, the azole class, particularly fluconazole (**5**), is the most commonly used treatment for various forms of candidiasis. However, resistance to fluconazole (**5**) and its associated side effects have become significant challenges in recent years [24,25], highlighting the urgent need for novel antimicrobial molecules with a wide range of pharmacological properties and reduced toxicity. Zidovudine (**6**), an approved oral antiviral drug, has emerged as one of the most repurposed antiviral drugs for combating multidrug-resistant Gram-negative bacterial infections. However, its therapeutic effect is hindered by the rapid development of bacterial resistance resulting from prolonged use, overuse, or misuse, underscoring the need for alternative therapeutic options [26]. Artesunic acid (**7**) is a semisynthetic derivative of artemisinin, a sesquiterpene extracted from *Artemisia annua* L., used for centuries to cure malaria in Chinese medicine. Research over the years has demonstrated the diverse potential of artemisinin compounds in various applications, including antitumor activity, immune regulation, osteoporosis prevention, and antibacterial and antiviral effects [27,28,29,30]. These findings have expanded the clinical utility of artemisinin, making it effective against a wide range of diseases. Ciprofloxacin, a fluoroquinolone antibiotic, has been used extensively for almost thirty years to treat many diseases, such as lower respiratory tract infections, urinary tract infections, gastric infections, endocarditis, skin infections, and chronic otorrhea. Its primary mechanism of action involves inhibiting DNA replication by targeting the A subunit of DNA gyrase while also influencing cell wall components. Ciprofloxacin (**8**) can be taken orally or intravenously, and it reaches therapeutic concentrations in the majority of body fluids and tissues; however, it is associated with potential adverse effects, including well-documented safety concerns and black box warnings related to tendon damage and other serious risks, particularly in specific patient populations [31,32,33].

**Scheme 1:**
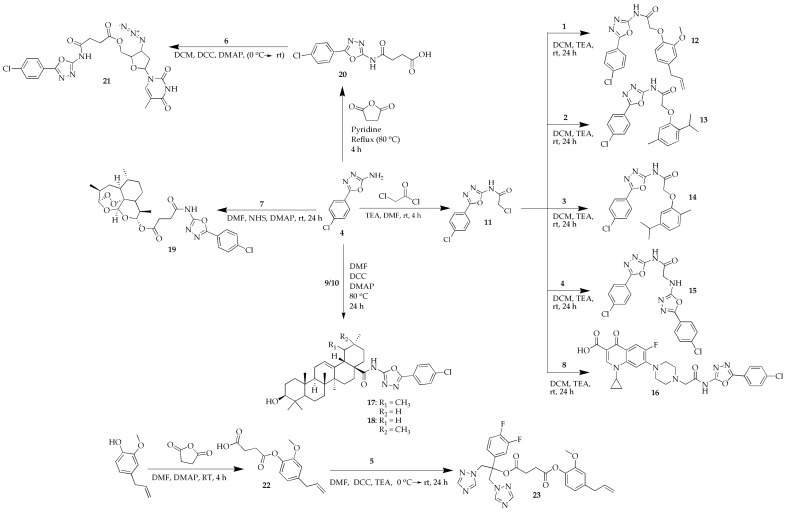
Synthesis of hybrid compounds (**12**–**19**, **21** and **23**).

Additionally, resistance to ciprofloxacin (**8**) has increasingly been reported in bacterial strains such as *E. coli*, *S. typhi*, *S. aureus*, and *P. aeruginosa* [34]. Oleanolic acid (**9**) and ursolic acid (**10**) are widely occurring isomeric triterpene phytochemicals known for their diverse pharmacological properties, such as anti-inflammatory, antitumor, antivirus, antibacterial, and antidiabetic properties [35,36,37,38]. However, their limited water solubility significantly restricts their clinical application [39]. We synthesized novel hybrid molecules **12**–**19**, **21,** and **23** directly linked with the abovementioned pharmaceutical scaffolds through esterification or amidation reactions or using linkers such as succinic anhydride or chloroacetyl chloride. Most of these hybrid molecules are linked with 5-(4-chlorophenyl)-1,3,4-oxadiazol-2-amine. Heterocyclic 1,3,4-oxadiazoles, known as effective bioisosteres for amides and esters, have the potential to interact with receptors through hydrogen bonding, which may significantly enhance their pharmacological activities [40]. The literature also highlights the potential pharmacological activities of oxadiazoles and their derivatives, such as antibacterial, anti-inflammatory, antifungal, and anticancer properties [40,41,42,43]. Based on all these reported findings, we synthesized and evaluated the antibacterial activity of the hybrid compounds, with most of them incorporating 5-(4-chlorophenyl)-1,3,4-oxadiazol-2-amine, as shown in Scheme 1.

## 2. Results and Discussion

### 2.1. Synthesis

#### 2.1.1. Synthesis of Hybrid Compounds **12**–**16**

The hybrid compounds, **12**–**16** were successfully synthesized through a two-step reaction sequence involving the chloroacetylation of 5-(4-chlorophenyl)-1,3,4-oxadiazol-2-amine (**4**) followed by nucleophilic substitution with bioactive molecules such as eugenol (**1**), thymol (**3**), carvacrol (**2**), and ciprofloxacin (**11**). These reactions proceeded with low to good yields, ranging from 29% to 60%. The structures of the synthesized hybrid molecules were confirmed using spectroscopic techniques, such as NMR, IR, and HRMS. The characteristic peaks corresponding to the functional groups provided strong evidence of successfully synthesizing the desired compounds. The eugenol-based hybrid compound **12** was obtained in a 57% yield with the presence of peaks at 1710 cm^−1^ (C=O stretching), 3554 cm^−1^ (N–H stretching), and 1626–1481 cm (C=C stretching) in the IR spectrum, confirming the formation of the amide linkage. The ^1^HNMR spectrum displayed signals at 7.81–7.79 ppm for aromatic protons, with peaks at 6.81–6.79 ppm and 5.33 ppm corresponding to the eugenol moiety. The ^13^C-NMR spectrum displayed a carbonyl carbon (C=O) at 171.16 ppm (C14), confirming the presence of an amide functional group. The peaks at 157.07 (C10) and 150.86 (C8) indicate electron-rich aromatic carbons, likely from the oxadiazole and eugenol moieties. The HRMS data further supported the proposed molecular formula. The thymol-based hybrid **13** was obtained with a 55% yield. A strong carbonyl absorption was visible at 1735 cm^−1^ in the IR spectrum, confirming the presence of the amide group. N–H stretching (3354 cm^−1^) suggested the presence of an amide. C=C stretching at 1500 cm^−1^ confirmed the presence of aromatic and alkene systems. The ^1^HNMR spectrum showed characteristic signals of thymol at 7.19–7.18 ppm and 6.37–6.35 ppm, confirming the presence of the oxadiazole and thymol moieties. The ^13^CNMR spectra indicated carbonyl carbon (C=O) at 1735 cm^−1^ and 170.31 ppm (C14), which confirmed the presence of an amide functional group. The peaks at 164.46 (C18) and 157.08 (C10) suggest electron-rich aromatic carbons from the oxadiazole and thymol moieties. The HRMS analysis provided a molecular ion peak that agrees with the calculated mass. Hybrid 14 containing carvacrol was synthesized with a 52% yield, slightly lower than the yield obtained for hybrid **13**. Hybrid **14** also demonstrated similar spectral characteristics with a C=O stretching band at 1694 cm^−1^ and N–H stretching at 3451 cm^−1^ in the IR spectrum. The ^1^HNMR spectrum exhibited signals corresponding to the carvacrol moiety, including peaks at 6.97–6.95 ppm and 6.56–6.54 ppm. The carbonyl carbon (C=O) at 171.21 ppm (C14) in the ^13^CNMR spectrum confirmed the presence of an amide functional group. The peaks at 164.48 (C18) and 157.07 (C10) indicated electron-deficient aromatic carbons, consistent with the oxadiazole and carvacrol moieties. The HRMS data validated the proposed structure. The synthesis of hybrid **15** was achieved with a relatively low yield of 29%, indicating potential challenges in the reaction efficiency or purification process. The ^13^C NMR spectrum confirmed the successful formation of the hybrid compound, with characteristic peaks for carbonyl (C=O) at 172.21 ppm and aromatic carbons ranging from 151.27 to 113.22 ppm. The ^1^H NMR spectrum further supported the structure, showing distinct signals for aromatic protons and the methylene group at δ 3.75 ppm, corresponding to the chloroacetyl moiety. The IR spectrum displayed key functional groups, including N–H stretching (3544–3352 cm^−1^), C=O stretching (1708 cm^−1^), and aromatic C=C stretching (1623–1493 cm^−1^), confirming the expected molecular framework. Additionally, the HRMS analysis provided a mass-to-charge ratio (*m*/*z*) of 430.0329, closely matching the calculated value of 430.0348, further validating the proposed structure. The low yield may be attributed to steric hindrance, incomplete conversion, or difficulties in purification, which could be optimized in future modifications. Hybrid **16** was successfully synthesized with a yield of 60%. The ^13^CNMR spectrum showed key signals for various carbon atoms, including the carbonyl (C=O) at 172.81 ppm, characteristic of the chloroacetyl group, and aromatic carbons ranging from 164.48 to 104.98 ppm, suggesting the presence of a complex aromatic system. The ^1^H NMR spectrum further confirmed the structure, with notable peaks such as a singlet at 9.02 ppm for the amide proton (H-4), doublets at 7.81–7.80 ppm and 7.61–7.59 ppm for aromatic protons, and additional singlets and multiplets corresponding to other hydrogen atoms in the structure. The IR spectrum showed significant functional groups, including OH stretching (3302 cm^−1^), C=O stretching (1688 cm^−1^), and aromatic C=C stretching (1667–1557 cm^−1^), which supported the presence of the anticipated functional groups. The HRMS analysis with a mass-to-charge ratio (*m*/*z*) of 566.1481 closely matched the calculated value (566.1521), confirming the molecular composition of hybrid **16**. Overall, the spectroscopic data support the successful synthesis of the hybrid compounds, with the yields indicating efficient reactions, although optimization may be considered to improve the yields further.

#### 2.1.2. Synthesis of Hybrid Compounds **17** and **18**

The obtained yields for both molecules were moderate, with 42% for compound **17** and 38% for compound **18**, indicating effective but not highly efficient conversions, likely due to steric hindrance or side reactions during amide bond formation. The structures of hybrids **17** and **18** were confirmed using various spectroscopic techniques. The IR spectra displayed characteristic peaks for NH stretching (3357 cm^−1^) and C=O stretching (1581 cm^−1^), confirming the amide bond formation. The ^1^HNMR spectra exhibited downfield shifts at 7.81 ppm (singlet, H-35) and 5.28 ppm (triplet, H-28) for hybrid **17**, and 7.95 ppm (singlet, H-36) and 5.14 ppm (triplet, H-29) for hybrid **18**, corresponding to aromatic and olefinic protons, respectively. The ^13^C NMR spectra further confirmed the structures with 172.31 ppm (C=O) peaks for hybrid **17** and 177.81 ppm (C=O) for hybrid **18**. The HRMS analysis validated the molecular compositions, with the observed masses matching the calculated values, indicating successful synthesis.

#### 2.1.3. Synthesis of Hybrid Compound **19**

Compound **19** was obtained with a 48% yield. The moderate yield could be attributed to possible steric effects or partial hydrolysis during purification. The IR spectrum exhibited NH stretching (3357 cm^−1^) and C=O stretching (1709 cm^−1^), confirming amide bond formation. The ^1^H NMR spectrum showed a characteristic singlet at 8.00 ppm (H-27), corresponding to the oxadiazole moiety, while the artesunate protons appeared in the range of 2.13–0.69 ppm. The ^13^CNMR data further supported the structural integrity, showing a characteristic carbonyl signal at 178.67 ppm (C26). HRMS confirmed the similarity of the calculated molecular weight (561.1878) with the observed value (561.1902), affirming successful hybridization.

#### 2.1.4. Synthesis of Hybrid Compound **21**

Hybrid **21**, a succinylated oxadiazole–zidovudine hybrid, was synthesized via a two-step process involving the formation of an N-succinylated intermediate (**20**) followed by coupling with zidovudine (**6**) using DCC/DMAP in dichloromethane. The reaction proceeded smoothly, yielding a final product that was isolated and purified using column chromatography. The IR analysis revealed NH stretching (3357 cm^−1^) and C=O stretching (1709 cm^−1^), supporting the formation of the desired conjugate. The ^1^H NMR spectrum displayed downfield shifts at 8.00 ppm (singlet, H-27), while the zidovudine (**6**) protons appeared in the expected region. The ^13^C NMR spectrum confirmed the presence of characteristic carbonyl and aromatic signals, consistent with the expected structure. The HRMS analysis validated the molecular weight, confirming the successful synthesis of hybrid **21**.

#### 2.1.5. Synthesis of Hybrid Compound **23**

Compound **23** was obtained with a yield of 53%, indicating moderate efficiency. The ^13^C NMR spectrum revealed key peaks for various carbon atoms, including the carbonyl (C=O) stretching at 172.86 ppm of the ester functional group, and signals for aromatic carbons in the range of 154.59 to 104.39 ppm. This confirmed the presence of the aromatic structures derived from eugenol (**1**) and fluconazole (**5**). The ^1^H NMR spectrum displayed characteristic signals, including a singlet at 8.33 ppm for the protons attached to the aromatic rings, as well as a complex multiplet in the range of 6.70–6.58 ppm, indicating the presence of several aromatic protons from the coupled components. The peaks for protons at 5.98–5.90 ppm and 5.04–5.03 ppm were likely due to the allyl and methoxy groups, further confirming the structure. The IR spectrum showed typical functional group vibrations, such as C=O stretching at 1710 cm^−1^, indicative of the ester group, and C=C stretching at 1471 cm^−1^, a characteristic aromatic group. The presence of C–O stretching signals between 1180 and 500 cm^−1^ also confirmed the ester linkage and the aromatic ring systems.

### 2.2. Antibacterial Evaluation

#### MIC

All the synthesized targeted compounds (**12**–**19**, **21**, and **23**) were assessed for their antibacterial potential against twelve bacterial strains, with DMSO as the negative control (Table 1). The most potent compounds against Gram-positive bacteria (*B. subtilis*, *E. faecalis*, *S. epidermidis*, *S. aureus*, and *M. smegmatis*) were compounds **12**–**16**, **19**, **21**, and **23**, which exhibited MIC values as low as 7.8125 µg/mL against various strains. Compound **16** was particularly noteworthy, as it exhibited MIC values of 7.8125 µg/mL for all the Gram-positive strains used in the study. Compounds **17** and **18** showed moderate activity against *E. faecalis* (MIC = 15.625 µg/mL), the most susceptible strain. Compound **16** again demonstrated the highest antibacterial activity, with an MIC of 7.8125 µg/mL against all Gram-negative bacteria. Compounds **12**–**16**, **19**, **21**, and **23** exhibited similar activities against Gram-negative strains, with MIC values of 7.8125 µg/mL against *E. cloacae*, *P. vulgaris*, *K. oxytoca*, and *P. aeruginosa*. Compounds **17** and **18** showed the highest activity with MIC values of 7.8125 and 15.625 µg/mL, respectively, against K. oxytoca, while compound **18** showed slightly higher MIC values, particularly against *E. cloacae*, *P. vulgaris*, *P. aeruginosa*, *P. mirabilis*, and *K. aerogenes* (31.25–62.5 µg/mL).

In view of the structure-activity relationship, the parent compounds used for hybridizing these compounds played an important role in their antibacterial activities. Among the tested hybrids, compound **16**, which incorporates ciprofloxacin via a chloroacetyl linker, demonstrated the most potent and broad-spectrum antibacterial activity. It exhibited MIC values of 7.81 µg/mL against all tested Gram-positive and Gram-negative bacterial strains, closely mirroring or surpassing the efficacy of the parent ciprofloxacin. This suggests that hybridization did not compromise the activity of ciprofloxacin but rather retained or enhanced its potency.

Hybrid compounds containing essential oil components, such as compound **12** containing eugenol, compound **13** containing thymol, compound **14** containing carvacrol, and compound **19** containing eugenol, exhibited the highest antibacterial activities, particularly against (*B. subtilis*, *E. faecalis*, *E. cloacae*, *P. vulgaris*, *P. aeruginosa*, and *K. oxytoca*) with MIC values of 7.8125 µg/mL. However, these compounds were less effective against *S. epidermidis*, *S. aureus*, *M. smegmatis*, *P. mirabilis*, *E. coli*, and *K. aerogenes*, where their MIC values ranged from 125 to 250 µg/mL. This variation in activity may be attributed to differences in the bacterial cell wall composition and membrane permeability [44].

Terpenoid-based compounds such as compound **17**, containing ursolic acid, and compound **18**, containing oleanolic acid, exhibited weaker antibacterial activity, with MIC values ranging from 125 to 250 µg/mL. Interestingly, the oleanolic acid-based hybrid compound **18** showed moderate activity against Gram-negative strains, such as *E. cloacae*, *P. vulgaris*, *K. oxytoca*, *P. aeruginosa*, and *P. mirabilis*, with MIC values ranging from 15.6 to 31.3 µg/mL, suggesting some degree of membrane penetration capability. However, these compounds were generally less effective against Gram-positive bacteria, likely due to steric hindrance and a lower interaction with bacterial targets [45,46].

Other hybrid compounds, such as the fluconazole-based compound **23** and zidovudine-based compound **21**, demonstrated similar antibacterial trends, effectively inhibiting *B. subtilis*, *E. faecalis*, *P. aeruginosa*, *P. vulgaris*, and *K. oxytoca* with MIC values of 7.81 µg/mL, while being less potent against *S. epidermidis*, *S. aureus*, *M. smegmatis*, and *K. aerogenes*. The dimer of 5-(4-chlorophenyl)-1,3,4-oxadiazol-2-amine (compound **15**) followed a similar pattern, reinforcing the notion that certain bacterial strains are more resistant to these compounds.

The antibacterial activities of hybrid compounds **12**–**19**, **21,** and **23** were compared to the standard ampicillin, streptomycin, and nalidixic acid. Among the tested compounds, compound **16** showed superior or equal activity to ciprofloxacin across all tested strains, making it the most potent hybrid. This activity was notably superior to the other standard drugs, particularly against resistant strains, where AMP, STM, and nalidixic acid showed much higher MIC values (e.g., NLD > 512 µg/mL against *E. faecalis* and *S. aureus*, STM = 512 µg/mL against *B. subtilis* and *K. aerogenes*). Compounds **12**–**15**, **19**, **21,** and **23** also displayed notable antibacterial activities, with MIC values ranging from 7.8125 to 250 µg/mL. In contrast, compounds **17** and **18** exhibited moderate antibacterial activities, with MIC values mostly between 15.6 and 250 µg/mL. Notably, compounds **12**–**15**, **19**, **21**, and **23** were more effective against Gram-negative bacteria (e.g., *E. cloacae*, *P. vulgaris*, *K. oxytoca*, and *P. aeruginosa*) compared to the standard antibiotics, particularly when compared to AMP and STM, which had significantly higher MIC values against these strains. Overall, this comparative analysis highlights the promising potential of ciprofloxacin-based and phenolic monoterpenoid-based hybrids as effective antibacterial agents, warranting further exploration and optimization.

According to the literature [47,48], the most prevalent bacterial strains in COVID-19 patients are *E. coli* and *K. pneumonia*. The current study demonstrated that compound **16**, the ciprofloxacin-based hybrid, exhibited the strongest activity against both *E. coli* and *K. pneumoniae*, with a MIC value of 7.81 µg/mL, far surpassing the activity of the standard ciprofloxacin (MIC = 250 µg/mL). This highlights the hybrid’s enhanced potency and potential for overcoming resistance mechanisms. All other hybrids, including those with natural monoterpenoids (eugenol, thymol, and carvacrol) and bioactive scaffolds like artesunic acid and zidovudine, demonstrated weak to moderate activity (MIC = 125–250 µg/mL), indicating limited effectiveness against these critical Gram-negative pathogens. The oleanolic acid hybrid (compound **18**) showed slightly improved activity against *K. pneumoniae* (MIC = 62.5 µg/mL) compared to its performance against *E. coli*, suggesting some scaffold-dependent variation in activity. Notably, standard antibiotics such as streptomycin and nalidixic acid showed high MIC values, reinforcing the challenge of treating *E. coli* and *Klebsiella* infections in the context of increasing resistance.

### 2.3. SwissADME Predictions

Drug-like chemical entities are extensively explored in drug discovery and candidate selection. Compounds with pharmacokinetic characteristics that enable them to remain viable throughout human clinical trials are classified as drug-like chemical species. The SwissADME database was utilized to assess the physicochemical properties, lipophilicity, water solubility, pharmacokinetic profile, drug-likeness, and medicinal chemistry of hybrid compounds **12**–**16**, **19**, **21,** and **23**.

#### 2.3.1. Physicochemical Properties

The physicochemical properties of a molecule play a crucial role in determining its efficacy, safety, and metabolism. These properties can be predicted using Lipinski’s rule of five, Veber’s rule, or Muegge’s rule [49]. The physicochemical properties of the hybrid compounds in Table 2 reveal distinct structural and molecular characteristics that influence their drug-like behaviours. Compounds **12**, **13**, **14**, and **15** have moderate molecular weights (ranging from 385.84 to 431.23 g/mol) and a balanced number of hydrogen bond acceptors (5–7) and donors (1–2), indicating a potential for good drug-like properties. Their molar refractivity values range from 104.62 to 106.19, and they exhibit total polar surface areas (TPSA) between 77.25 and 118.97 Å^2^, which suggests a reasonable balance between permeability and solubility. In contrast, compounds **16**, **19**, **21**, and **23** have significantly higher molecular weights (above 540 g/mol), exceeding Lipinski’s rule for optimal oral bioavailability. These compounds also have higher numbers of heavy atoms (38–40) and rotatable bonds (8–15), which may negatively affect their conformational flexibility and bioavailability. Compound 21 has an exceptionally high TPSA (208.16 Å^2^), suggesting poor membrane permeability. Moreover, compounds **19** and **23** possess a higher fraction of sp^3^-hybridized carbons (0.63 and 0.26, respectively), which may influence their three-dimensional complexity. Overall, while compounds **12**–**15** exhibit more favourable physicochemical properties for drug-likeness, compounds **16**, **19**, **21**, and **23** may require modifications to improve their bioavailability and permeability.

#### 2.3.2. Lipophilicity

As shown in Table 3, the lipophilicity of the hybrid compounds, as measured by their consensus Log P_o/w_ values, reveals variations in their hydrophobicity, which directly impacts their absorption, distribution, and bioavailability. Compounds **12**, **13**, **14**, and **15** exhibit moderate lipophilicity, with consensus Log P_o/w_ values ranging from 3.26 to 4.12. Among these, compounds **13** and **14** have the highest lipophilicity (4.11 and 4.12, respectively), suggesting good membrane permeability but a potential risk of reduced aqueous solubility. Compound **16** has the lowest lipophilicity (Log P_o/w_ = 2.89), indicating better solubility but potentially lower membrane permeability. Meanwhile, compound **19** shows relatively high lipophilicity (Log P_o/w_ = 3.93), suggesting favourable permeability characteristics. In contrast, compound **21** has the lowest lipophilicity (Log P_o/w_ = 1.69), which may lead to challenges in crossing lipid membranes and reduced oral bioavailability. Compound **23** falls in the moderate range (Log P_o/w_ = 3.69), suggesting balanced permeability and solubility. Overall, while most compounds have lipophilicity values within an acceptable range for drug-likeness, compounds with very high or very low Log P_o/w_ values may require further modification to enhance their pharmacokinetic properties.

#### 2.3.3. Water Solubility

The water solubility of the hybrid compounds varies across different prediction methods. Based on the ESOL model, compounds **12**, **13**, **14**, **15**, **16**, **19**, and **23** exhibit moderate solubility, with solubilities ranging from 7.77 × 10^−4^ mg/mL to 7.69 × 10^−3^ mg/mL, as shown in Table 4. Compound **21**, however, is classified as soluble, with a solubility of 1.12 × 10^−1^ mg/mL. According to the Ali model, the solubility trend is similar, with most compounds classified as moderately soluble, except for compound **21**, which remains moderately soluble but with a lower value of 2.47 × 10^−3^ mg/mL. In the SILICOS-IT model, most of the compounds are poorly soluble, with solubility values ranging from 1.46 × 10^−6^ mg/mL to 1.48 × 10^−5^ mg/mL. Compound **21**, however, shows moderate solubility, at 4.11 × 10^−4^ mg/mL. Overall, solubility predictions indicate that while most hybrids are moderately soluble, their solubility decreases significantly when evaluated through the SILICOS-IT model.

#### 2.3.4. Pharmacokinetics

In Table 5, the pharmacokinetics of the hybrid compounds indicate diverse absorption, permeability, and metabolic profiles. Compounds **12**, **13**, **14**, **15**, and **23** demonstrate high gastrointestinal (GI) absorption, suggesting good systemic availability upon oral administration. These compounds also show the potential to inhibit several cytochrome P450 enzymes, including CYP1A2, CYP2C19, CYP2C9, and CYP3A4, which could influence the metabolism of co-administered drugs. However, none of these compounds are substrates for P-glycoprotein (P-gp), and most are not permeable to the blood–brain barrier (BBB), which limits their potential central nervous system effects. In contrast, compounds **19** and **21** exhibit lower GI absorption, with compound **19** showing reduced permeability (low GI absorption, BBB non-permeant, and P-gp substrate). These compounds also exhibit different inhibition profiles, with compound **16**, in particular, showing broader inhibitory activity toward various CYP enzymes (CYP2C19, CYP2C9, CYP2D6, and CYP3A4). Skin permeation, as indicated by Log Kp values, was also evaluated, with all compounds showing low skin permeability, ranging from −5.69 cm/s to −8.65 cm/s, suggesting that they may not be suitable for transdermal delivery.

#### 2.3.5. Drug-Likeness

The drug-likeness of the hybrid compounds in Table 6 reveals a range of profiles based on different drug-likeness rules. Compounds **12**, **13**, **14**, and **15** all adhere to Lipinski’s rule of five, with no violations, suggesting they possess good oral bioavailability potential. These compounds also pass the Ghose, Veber, Egan, and Muegge filters, further supporting their drug-likeness. These compounds show a relatively high bioavailability score of 0.55, indicating moderate to good bioavailability. However, compounds **16**, **19**, **21**, and **23** display significant deviations from the drug-likeness rules. For example, compound **16** violates Lipinski’s rule with a molecular weight (MW) >500 and the number of rotatable bonds (NorO) >10 and violates Muegge’s criteria with a topological polar surface area (TPSA) >131.6. Compound **19** exhibits more significant violations in terms of MW, molecular rigidity (MR), and atom count. Compounds **21** and **23** also show multiple violations, including TPSA and hydrogen bond acceptors (H-acc), resulting in lower bioavailability scores (0.17). Overall, while compounds **12**–**15** demonstrate strong drug-likeness, others may require further modification to improve their drug-like properties.

#### 2.3.6. Medicinal Chemistry

The medicinal chemistry analysis of the hybrid compounds demonstrated in Table 7 indicates varying profiles in terms of potential alerts, lead likeness, and synthetic accessibility. Compounds **12**, **13**, **14**, and **15** show no PAINS (pan-assay interference compounds) alerts, indicating that they do not contain functional groups likely to interfere with assay results. These compounds also demonstrate relatively favourable synthetic accessibility, with compound **15** showing the lowest accessibility score (3.12), suggesting that it may be slightly more challenging to synthesize. Compounds **16**, **19**, **21**, and **23**, however, exhibit more complex profiles. Compound **16** has no PAINS alerts but shows two violations related to molecular weight (MW) and rotatable bonds. Compound **19** contains a peroxide alert, which could pose stability issues, and it has a higher synthetic accessibility score (7.12). Compound **21** features multiple PAINS alerts, including azo and diazo groups, which could reduce its drug-likeness and increase its synthetic complexity (accessibility score of 5.03). Compound **23** has alerts related to isolated alkenes and phenol esters, which may affect its stability and reactivity, and it has a moderate synthetic accessibility score (3.97). Overall, compounds **12**–**15** are more favourable in terms of medicinal chemistry, while the others may require careful optimization to address their alerts and synthetic challenges.

## 3. Materials and Methods

### 3.1. Materials

All reactions were carried out using anhydrous solvents and reagents purchased from Sigma-Aldrich, Merck Pty Ltd., Johannesburg, South Africa), and were used without further purification. All reactions were performed under a nitrogen atmosphere. The synthesized hybrid molecules were characterized using ^1^H NMR and ^13^C NMR spectroscopy, Fourier Transform Infrared Spectroscopy (FTIR), and High-Resolution Mass Spectrometry (HRMS). NMR spectra were recorded using a Bruker Avance III 500 MHz.

NMR spectrometer (Bruker BioSpin GmbH, Rheinstetten, Germany), with residual solvent peaks as internal references. NMR spectra were recorded using a Bruker Avance III 500 MHz NMR spectrometer (Bruker BioSpin GmbH, Rheinstetten, Germany), with residual solvent peaks as internal references. Chemical shifts (δ) are reported in parts per million (ppm). FTIR spectra were obtained using a PerkinElmer Spectrum Two FTIR spectrometer (PerkinElmer Inc., Waltham, MA, USA), while HRMS data were acquired using a Thermo Scientific Q Exactive Orbitrap Mass Spectrometer (Thermo Fisher Scientific, Waltham, MA, USA).

Thin-layer chromatography (TLC) was performed on silica gel 60 F254 aluminium-backed plates (Merck KGaA, Darmstadt, Germany). Compounds were visualized using UV light at both 365 nm (long wavelength) and 254 nm (short wavelength), and with vanillin–sulfuric acid spray reagent (prepared from 100 mL methanol, 1 g vanillin, and 0.5 mL sulfuric acid; all from Sigma-Aldrich, Merck Pty Ltd., Johannesburg, South Africa). The synthesized compounds were purified using column chromatography on silica gel 60 (230–400 mesh ASTM, 0.04–0.063 mm particle size) (Merck KGaA, Darmstadt, Germany). Data processing and spectral analysis were performed using MestReNova software (version 14.2.1).

### 3.2. Chemistry

#### 3.2.1. General Synthetic Procedure for Hybrid Compounds **12**–**16**

Bulleted compounds **12***–***16** were synthesized by following the procedure outlined in Scheme 1 using a modified version of the synthetic procedures established by Anthwa et al. [50], Salama et al. [51], and Ayyash et al. [52]. 5-(4-Chlorophenyl)-1,3,4-oxadiazol-2-amine (**4**) (1 g, 5.11 mmol, 1 equivalent) was reacted with chloroacetyl chloride (0.577 g, 5.11 mmol, 1 equivalent) in the presence of TEA (0.85 mL, 6.13 mmol, 1.2 equivalents) using anhydrous DCM (25 mL) as a solvent to form N-(chloroacetyl)-5-(4-chlorophenyl)-1,3,4-oxadiazol-2-amine (**11**). The base (TEA) neutralized the hydrochloric acid formed during the reaction. The resulting chloroacetylated intermediate (**11**) was then reacted with compounds **1***–***4** and **8** in the presence of TEA to form the final products **12***–***16** through nucleophilic attack by the hydroxyl groups of eugenol (**1**), thymol (**3**), carvacrol (**2**) or by the NH group of 5-(4-chlorophenyl)-1,3,4-oxadiazol-2-amine or ciprofloxacin on the electrophilic chloroacetyl group. After each reaction step, the organic phase was washed with brine and water, dried using Na_2_SO_4_, and evaporated under low pressure. The final products were then purified by column chromatography.

2-(4-Allyl-2-methoxyphenoxy)-N-(5-(4-chlorophenyl)-1,3,4-oxadiazol-2-yl)acetamide (**12**)**:** N-(chloroacetyl)-5-(4-chlorophenyl)-1,3,4-oxadiazol-2-amine (**11**) (0.200 g, 0.732 mmol, 1.0 equivalent), eugenol (**1**) (0.120 g, 0.732 mmol, 1.0 equivalent), TEA (0.12 mL, 6.13 mmol, 1.2 equivalent), DCM(20 mL). Column eluent: EtOAc/Hex(4:10). Yield: 57%. R_f_ = 0.56.

^13^C-NMR (500 MHz, recorded in DMSO-d_6_): chemical shifts (δ, ppm)—171.16 (C-1, 157.07 (C-10), 150.86 (C-8), 136.49 (C-23), 135.37 (C-18), 129.81 (C-1, C-3), 127.22 (C-4, C-5, C-6), 123.75 (C-21), 109.96 (C-20), 84.49 (C-19), 83.96 (C-28), 61.32 (C-22), 60.67 (C-15), 36.68 (C-25), 12.62 (C-26) (Figure S1).

^1^H-NMR (500-MHz, recorded in DMSO-d_6_): ppm(δ) = 7.80 (H-6, doublet, 1H, *J* = 10.0 Hz), 7.80 (H-4, doublet, 1H, *J* = 10.0 Hz), 7.59 (H-1, doublet, 1H, *J* = 10.0 Hz), 7.59 (H-3, doublet, 1H, *J* = 10.0 Hz), 7.28 (H-22, singlet, 1H), 7.21 (H-20, doublet, 1H, *J* = 5.0 Hz), 6.80 (H-19, doublet, 1H, *J* = 10.0), 6.68–6.58 (H-27, multiplet, 1H), 5.33 (H-13, singlet, 1H), 4,85 (H-15, singlet, 1H), 4.42–4.39 (H-28, doublet of a doublet, 2H), 3.32 (H-25, singlet, 3H), 3.10 (H-26, doublet, 1H, *J* = 10.0 Hz) (Figure S1).

FT–IR (KBr ν in cm^−1^): 3554 (N–H stretching), 2931–2855 (C–H stretching from alkanes), 1710 (C=O stretching), 1626–1481 (C=C stretching from aromatic or alkene), 1381–1156 (C–H bending (alkanes or aromatics)), 1000–500 (C–O stretching, C–Cl bonds, or skeletal vibrations from aromatic rings) (Figure S11).

HRMS (ESI) mass spectrometry analysis (*m*/*z*) of [C_20_H_18_ClN_3_O_4_]^+^: calculated 399.0986, observed 399.0958 (Figure S21).

2-(2-Isopropyl-5-methylphenoxy)-N-(5-(4-chlorophenyl)-1,3,4-oxadiazol-2-yl)acetamide (**13**): N-(chloroacetyl)-5-(4-chlorophenyl)-1,3,4-oxadiazol-2-amine (**11**) (0.200 g, 0.732 mmol, 1.0 equivalent), thymol (**3**) (131.9 mg, 0.878 mmol, 1.2 equivalents), TEA (0.153 mL, 1.098 mmol, 1.5 equivalents). Column eluent: EtOAc/Hex (7:3). Yield: 55%. R_f_ = 0.58.

^13^C-NMR (500 MHz, recorded in DMSO-d_6_): chemical shifts (δ, ppm)—170.31 (C-14), 164.46 (C-18), 157.08 (C-10), 155.09 (C-8), 136.50 (C-2), 135.39 (C-20), 134.46 (C-23), 129.79 (C-3), 129.05 (C-4), 127.23 (C-22), 123.69 (C-21), 115.86 (C-19), 68.09 (C-15), 25.97 (C-24), 23.06 (C-27), 22.36 (C-25), 21.72 (C-26) (Figure S2).

^1^H-NMR (500 MHz, recorded in DMSO-d_6_): chemical shifts (δ, ppm)—7.80 (H-6, doublet, 1H, *J* = 10.0 Hz), 7.80 (H-4, doublet, 1H, *J* = 10.0 Hz), 7.58 (H-1, doublet, 1H, *J* = 10.0 Hz), 7.58 (H-3, doublet, 1H, *J* = 10.0 Hz), 7.28 (H-19, singlet, 1H), 7.19 (H-22, doublet, 1H, *J* = 5.0 Hz), 6.37 (H-21, doublet, 1H, *J* = 10.0 Hz), 5.73 (H-13, singlet, 1H), 4.62 (H-15, singlet, 2H), 3.69–3.58 (H-24, multiplet, 1H), 3.39 (H-25, singlet, 3H), 1.10 (H-27, doublet, 6H, *J* = 10.0 Hz) (Figure S2).

FT–IR (KBr, ν in cm^−1^): 3354 (N–H stretching), 2933–2872 (C–H stretching from alkanes), 1735 (C=O stretching), 1500 (C=C stretching from aromatic or alkene), 1388 (C–H bending of alkanes or aromatics), 1157–500 (C–O stretching, C–Cl bond, or skeletal vibrations from aromatic rings) (Figure S12).

HRMS (ESI) mass spectrometry analysis (*m*/*z*) of [C_20_H_20_ClN_3_O_3_]^+^: calculated 385.1193, observed 385.1185 (Figure S22).

2-(5-Isopropyl-2-methylphenoxy)-N-(5-(4-chlorophenyl)-1,3,4-oxadiazol-2-yl)acetamide (**14**): N-(chloroacetyl)-5-(4-chlorophenyl)-1,3,4-oxadiazol-2-amine (**11**) (0.200 g, 0.732 mmol, 1.0 equivalent), carvacrol (**2**) (131.9 mg, 0.878 mmol, 1.2 equivalents), TEA (0.153 mL, 1.098 mmol, 1.5 equivalents). Column eluent: EtOAc/Hex (3:7). Yield: 52%. R_f_ = 0.63.

^13^C-NMR (500 MHz, recorded in DMSO-d_6_): chemical shifts (δ, ppm)—171.21 (C-14), 164.48 (C-18), 157.07 (C-10), 154.61 (C-8), 135.71 (C-3), 135.38 (C-1), 131.68 (C-22), 129.82 (C-5), 127.23 (C-4), 126.08 (C-6), 123.75 (C-23), 120.10 (C-21), 116.06 (C-19), 71.02 (C-15), 26.51 (C-25), 23.05 (C-26), 23.05 (C-27), 21.10 (C-24) (Figure S3).

^1^H-NMR (500 MHz, recorded in DMSO-d_6_): chemical shifts (δ, ppm)—7.81 (H-6, doublet, 1H, *J* = 5.0 Hz), 7.81 (H-4, doublet, 1H, *J* = 5.0 Hz), 7.60 (H-1, doublet, 1H, *J* = 5.0 Hz), 7.60 (H-3, doublet, 1H, *J* = 5 Hz), 6.97–6.95 (H-22, doublet, 1H, *J* = 10.0 Hz), 6.56 (H-19, singlet, 1H), 6.55 (H-21, doublet, 1H, *J* = 10.0 Hz), 4.88 (H-13, singlet, 1H), 3.33 (H-15, singlet, 1H), 3.18–3.12 (H-25, multiplet, 1H), 2.17 (H-24, doublet, 3H, *J* = 5.0 Hz), 1.13 (H-26, doublet, 3H, *J* = 10.0 Hz), 1.13 (H-27, doublet, 3H, *J* = 10.0 Hz) (Figure S3).

FT–IR (KBr, ν in cm^−1^): 3451 (N–H stretching), 2942–2890 (C–H stretching from alkanes), 1694 (C=O stretching), 1468–1391 (C=C stretching from aromatic or alkene). 1389–1033 (C–H bending (alkanes or aromatics)), 1000–500 (C–O stretching, C–Cl bonds, or skeletal vibrations from aromatic rings) (Figure S13).

HRMS (ESI) mass spectrometry analysis analysis (*m*/*z*) of [C_20_H_20_ClN_3_O_3_]^+^: calculated 385.1193, observed 385.1179 (Figure S23).

2-(5-(4-Chlorophenyl)-1,3,4-oxadiazol-2-ylamino)-N-(5-(4-chlorophenyl)-1,3,4-oxadiazol-2-yl)acetamide (**15**): N-(chloroacetyl)-5-(4-chlorophenyl)-1,3,4-oxadiazol-2-amine (**11**) (0.300 g, 1.098 mmol, 1.0 equivalent), 5-(4-chlorophenyl)-1,3,4-oxadiazol-2-amine (260.5 mg, 1.318 mmol, 1.2 equivalents), TEA (0.23 mL, 1.647 mmol, 1.5 equivalents). Column eluent: EtOAc/Hex (3:5). Yield: 29%. R_f_ = 0.25.

^13^C-NMR (500 MHz, recorded in DMSO-d_6_): chemical shifts (δ, ppm)—172.21 (C-14), 151.27 (C-26), 147.97 (C-10), 145.58 (C-8), 145.24 (C-24), 138.66 (C-19), 130.95 (C-21), 121.02 (C-5), 115.90 (C-6), 115.67 (C-22), 113.22 (C-20), 56.09 (C-15) (Figure S4).

^1^H-NMR (500 MHz, recorded in DMSO-d_6_): chemical shifts (δ, ppm) = 6.73 (H-4, doublet, 1H, *J* = 10.0 Hz), 6.73 (H-6, doublet, 1H, *J* = 10.0 Hz), 6.70 (H-22, doublet, 1H, *J* = 5.0 Hz), 6.70 (H-20, doublet, 1H, *J* = 5.0 Hz), 6.58 (H-1, doublet, 1H, *J* = 10.0 Hz), 6.58 (H-3, doublet, 1H, *J* = 10.0 Hz), 6.58 (H-17, doublet, 1H, *J* = 10.0 Hz), 6.58 (H-19, doublet, 1H, *J* = 10.0 Hz), 5.08 (H-13, singlet, 1H), 3.75 (H-15, doublet, 2H, *J* = 10.0 Hz), 3.32 (H-29, singlet, 1H) (Figure S4).

FT–IR (KBr, ν in cm^−1^): 3544–3352 (N–H stretching) 2930–2856 (C–H stretching from alkanes), 1708 (C=O stretching), 1623–1493 (C=C stretching (aromatic or alkene)), 1455–1361 (C–H bending (alkanes or aromatics)), 1156–500 (C–O stretching, C–Cl bonds, or skeletal vibrations from aromatic rings) (Figure S14).

HRMS (ESI) mass spectrometry analysis (*m*/*z*) of [C_18_H_12_Cl_2_N_6_O_3_]^+^: calculated 430.0348, observed 430.0329 (Figure S24).

7-(4-(2-(5-(4-Chlorophenyl)-1,3,4-oxadiazol-2-ylamino)acetyl)piperazin-1-yl)-1-cyclopropyl-6-fluoro-1,4-dihydro-4-oxoquinoline-3-carboxylic acid (**16**): N-(chloroacetyl)-5-(4-chlorophenyl)-1,3,4-oxadiazol-2-amine (200 mg, 0.732 mmol, 1.0 equivalent), ciprofloxacin (290.7 mg, 0.878 mmol, 1.2 equivalents), TEA (0.153 mL, 1.098 mmol, 1.5 equivalents). Column eluent: EtOAc/Hex 2:8. Yield: 60%. R_f_ = 0.62.

^13^C-NMR (500 MHz, recorded in DMSO-d_6_): chemical shifts (δ, ppm)—172.81 (C-22), 164.48 (C-25), 157.07 (C-2), 154.61 (C29), 148.72 (C-31), 147.30 (C-12), 145.79 (C-14), 135.71 (C-4), 135.38 (C-37), 131.68 (C-9), 129.82 (C38), 127.23 (C-38), 126.08 (C-36), 123.75 (C-34), 120.10 (C-39), 116.06 (C-35), 110.99 (C-10), 107.99 (C-11), 104.98 (C-3), 53.79 (C-21), 52.89 (C-16), 47.45 (C-20), 46.33 (C-19), 43.14 (C-17), 26.51 (C-26), 23.95 (C-6), 23.95 (C-7), 21.10 (C-8) (Figure S5).

^1^H-NMR (500 MHz, recorded in DMSO-d_6_): chemical shifts (δ, ppm)—8.33 (H-4, singlet, 1H), 7.49 (H-34, doublet, 1H, *J* = 5.0 Hz), 7.49 (H-38, doublet, 1H, *J* = 5.0 Hz), 7.45 (H-35, doublet, 1H, *J* = 10.0 Hz), 7.43 (H-37, singlet, 1H), 6.35 (H-21, singlet, 1H, *J* = 5.0 Hz), 5.36 (H-27, singlet, 1H), 3.73 (H-20, triplet, 2H, *J* = 5.0 Hz), 3.51 (H-16, triplet, 2H, *J* = 5.0 Hz), 3.23 (H-25, singlet, 2H), 3.01–2.94 (H-6, multiplet, 1H), 2.79 (H-19, triplet, 2H, *J* = 5.0 Hz), 2.66 (H-17, triplet, 2H, *J* = 5.0 Hz), 0.88–0.77 (H-7, multiplet, 2H), 0.77–0.67 (H-8, multiplet, 2H) (Figure S5).

FT-IR (KBr, ν in cm^−1^): 3302 (OH stretching), 2942–2891 (C–H stretching from alkanes), 1688 (C=O stretching), 1667–1557 (C=C stretching from aromatic or alkene), 1465–1373 (C–H bending (alkanes or aromatics)), 1043–500 (C–O stretching, C–Cl bonds, or skeletal vibrations from aromatic rings) (Figure S15).

HRMS (ESI) mass spectrometry analysis (*m*/*z*) of [C_27_H_24_ClFN_6_O_5_]^+^: calculated 566.1481, observed 566.1521 (Figure S25).

#### 3.2.2. General Synthetic Procedure for Hybrid Compounds **17** and **18**

Two ursolic acid or oleanolic acid derivatives containing the 5-(4-chlorophenyl)-1,3,4-oxadiazol-2-amine moiety were synthesized as outlined in Scheme 1 by following a modified procedure [53]. UA or OA was dissolved in anhydrous DMF, and then DCC and DMAP were added to activate their carboxyl groups. 5-(4-Chlorophenyl)-1,3,4-oxadiazol-2-amine (**4**) was then introduced, allowing the amine group to react with the activated carboxyl group of ursolic acid or oleanolic acid, forming an amide bond. The reaction mixture was stirred at room temperature for 1 h and refluxed at 80 °C overnight. After the reaction, the solvent was removed, and the product was purified using column chromatography.

Hybrid **17**: ursolic acid (300 mg, 0.657 mmol, 1.0 equivalent), 5-(4-chlorophenyl)-1,3,4-oxadiazol-2-amine (155.2 mg, 0.789 mmol, 1.2 equivalents), DCC (149.2 mg, 0.723 mmol, 1.1 equivalents), DMAP (8.1 mg, 0.066 mmol, 0.1 equivalent), DMF (10 mL). Column eluent: EtOAc/Hex (3:7). Yield: 42%. R_f_ = 0.72.

^13^C-NMR (500 MHz, recorded in CDCl-d_3_): chemical shifts (δ, ppm)—172.31 (C-1), 157.09 (C-36), 156.02 (C-39), 138.33 (C-27), 136.07 (C-44), 132.15 (C-45), 130.67 (C-43), 128.77 (C-41), 127.60 (C-46), 125.96 (C-42), 124.54 (C-28), 77.29 (C-2), 55.22 (C-9), 52.41 (C-25), 49.65 (C-30), 47.98 (C-18), 47.35 (C-14), 42.13 (C-12), 39.08 (C-23), 38.85 (C-6), 38.66 (C-21), 36.95 (C-5), 33.81 (C-31), 31.62 (C-11), 30.31 (C-20), 29.45 (C-4), 28.70 (C-15), 25.80 (C-29), 24.92 (C-7), 23.76 (C-8), 21.24 (C-10), 17.46 (C-22), 17.37 (C-24), 16.52 (C-13), 15.50 (C-32) (Figure S6).

^1^H-NMR (500 MHz, recorded in DMSO-d_6_): chemical shifts (δ, ppm)—7.97 (H-33, singlet, 1H), 7.71 (H-41, doublet, 1H, *J* = 5.0 Hz), 7.71 (H-45, doublet, 1H, *J* = 5.0 Hz), 7.66 (H-42, doublet, 1H, *J* = 5.0 Hz), 7.66 (H-44, doublet, 1H, *J* = 10.0 Hz), 5.40 (H-48, triplet, 1H, *J* = 5.0 Hz), 3.01 (H-2, triplet, 1H, *J* = 5.0 Hz), 2.12 (H-44, doublet, 1H, *J* = 10.0 Hz), 1.93–0.69 (aliphatic protons from the ursolic acid moiety) (Figure S6).

FT-IR (KBr, ν in cm^−1^): 3357 (NH stretching), 3265 (OH stretching), 2941–2871 (C–H stretching from alkanes), 1581 (C=O stretching), 1557–1436 (C=C stretching from aromatic or alkene), 1375–1138 (C–H bending (alkanes or aromatics)), 902–500 (C–O stretching, C–Cl bonds, or skeletal vibrations from aromatic rings) (Figure S16).

HRMS (ESI) mass spectrometry analysis (*m*/*z*) of [C_38_H_52_Cl_2_N_3_O_3_]^+^: calculated 633.3697, observed 633. 3665 (Figure S26).

Hybrid **18**: oleanolic acid (300 mg, 0.657 mmol, 1.0 equivalent), 5-(4-chlorophenyl)-1,3,4-oxadiazol-2-amine (155.2 mg, 0.789 mmol, 1.2 equivalents), DCC (149.2 mg, 0.723 mmol, 1.1 equivalents), DMAP (8.1 mg, 0.066 mmol, 0.1 equivalent). Column eluent: EtOAc/Hex (4:6). Yield: 38%. R_f_ = 0.3.

^13^C-NMR (500 MHz, recorded in DMSO-d_6_): chemical shifts (δ, ppm)—177.81 (C-1), 157.13 (C-34), 154.08 (C-2), 140.33 (C-25), 136.56 (C-42), 134.34 (C-43), 134.08 (C-41), 129.25 (C-39), 126.93 (C-44), 126.93 (C-40), 80.39 (C-2), 52.53 (C-9), 51.58 (C-28), 45.47 (C-22), 44.37 (C-18), 40.85 (C-23), 37.47 (C-14), 37.36 (C-12), 36.39 (C-6), 36.30 (C-5), 34.73 (C-29), 34.22 (C-20), 30.81 (C-11), 26.04 (C-21), 25.92 (C-46), 25.92 (C-47), 24.72 (C-19), 24.55 (C-19), 20.35 (C16), 13.17 (C-13), 12.72 (C-30) (Figure S7).

^1^H-NMR (500 MHz, recorded in DMSO-d_6_): chemical shifts (δ, ppm)—7.95 (H-36, singlet, 1H), 7.03 (H-43, doublet, 1H, *J* = 10.0 Hz), 7.03 (H-47, doublet, 1H, *J* = 10.0 Hz), 6.67 (H-44, doublet, 1H, *J* = 10.0 Hz), 6.67 (H-46, doublet, 1H, *J* = 10.0 Hz), 5.14 (H-29, triplet, 1H, *J* = 5.0 Hz), 3.0 1 (H-27, triplet, 1H, *J* = 5.0 Hz), 2.48–2.47 (H-2, doublet of a doublet, 1H, *J* = 5.0 Hz), 2.21–0.69 (aliphatic protons from the oleanolic acid moiety) (Figure S7).

FT-IR (KBr, ν in cm^−1^): 3357 (NH stretching), 3265 (OH stretching), 2941–2871 (C–H stretching from alkanes), 1581 (C=O stretching), 1557–1436 (C=C stretching from aromatic or alkene), 1375–1138 (C–H bending (alkanes or aromatics)), 902–500 (C–O stretching, C–Cl bonds, or skeletal vibrations from aromatic rings) (Figure S17).

HRMS (ESI) mass spectrometry analysis (*m*/*z*) of [C_38_H_52_Cl2N_3_O_3_]^+^: calculated 633.3697, observed 633.3635 (Figure S27).

#### 3.2.3. Synthetic Procedure for Hybrid Compound **19**

The synthesis of a 5-(4-chlorophenyl)-1,3,4-oxadiazol-2-amine-artesunate hybrid (**19**) was carried out by activating artesunic acid (**7**) to react with the amine group of 5-(4-chlorophenyl)-1,3,4-oxadiazol-2-amine. First, artesunic acid (**7**) was dissolved in anhydrous DMF and activated using NHS to form an ester. Next, the activated artesunate was reacted with the amine group of 5-(4-chlorophenyl)-1,3,4-oxadiazol-2-amine in DMF at room temperature, and the reaction was allowed to proceed for 24 h. After completion, the solvent was removed, and the crude product was extracted with dichloromethane and purified with column chromatography.

Artesunic acid (250 mg, 0.621 mmol, 1.0 equivalent), 5-(4-chlorophenyl)-1,3,4-oxadiazol-2-amine (122.1 mg, 0.261 mmol, 1.0 equivalent), NHS (78.6 mg, 0.683 mmol, 1.1 equivalent). Column eluent: EtOAc/Hex (3:7). Yield: 48%. R_f_ = 0.47.

^13^C-NMR (500 MHz, recorded in DMSO-d_6_): chemical shifts (δ, ppm)—178.67 (C-26), 173.18 (C-22), 166.56 (C-32), 164.30 (C-29), 138.67 (C-37), 132.25 (C-38), 129.85 (C-36), 126.83 (C-34), 125.05 (C-39), 123.97 (C-35), 77.34 (C-5), 55.27 (C-6), 52.89 (C-11), 47.32 (C-18), 42.32 (C-1), 38.97 (C-13), 38.91 (C-14), 36.79 (C-25), 30.38 (C-7), 28.73 (C-24), 23.74 (C-12), 21.50 (C-15), 16.51 (C-20), 15.68 (C-8) (Figure S8).

^1^H-NMR (500 MHz, recorded in DMSO-d_6_): chemical shifts (δ, ppm)—8.00 (H-27, singlet, 1H), 7.99 (H-35, doublet, 1H, *J* = 5.0 Hz), 7.99 (H-39, doublet, 1H, *J* = 5.0 Hz), 7.63 (H-36, doublet, 1H, *J* = 5.0 Hz), 7.60 (H-38, doublet, 1H, *J* = 5.0 Hz), 6.04 (H-19, doublet, 1H, *J* = 5.0 Hz), 5.14 (H-2, doublet, 1H, *J* = 5.0 Hz), 4.26 (H-41, singlet, 1H), 3.15 (H-24, triplet, 2H, *J* = 5.0 Hz), 2.82 (H-25, triplet, 2H, *J* = 5.0 Hz), 2.13–0.69 (aliphatic protons from artesunate) (Figure S8).

FT–IR (KBr, ν in cm^−1^): 3357 (NH stretching), 3550–3371 (NH stretching), 2930–2858 (C–H stretching from alkanes), 1709 (C=O stretching), 1627 (C=C stretch from aromatic or alkene), 1383–1209 (C–H bending (alkanes or aromatics)), 1035–500 (C–O stretching, C–Cl bonds, or skeletal vibrations from aromatic rings) (Figure S18).

HRMS (ESI) mass spectrometry analysis (*m*/*z*) of [C_27_H_32_ClN_3_O_8_]^+^: calculated 561.1878, observed 561.1902 (Figure S28).

#### 3.2.4. Synthetic Procedure for Hybrid Compound **21**

5-(4-Chlorophenyl)-1,3,4-oxadiazol-2-amine (250 mg, 1.272 mmol, 1.0 equivalent) was reacted with succinic anhydride (152.7 mg, 1.527 mmol, 1.2 equivalents) in pyridine under reflux. After 4 h, the product was precipitated in cold water, filtered, and dried to yield the N-succinylated intermediate (**20**). The N-succinylated intermediate was dissolved in dichloromethane, followed by the addition of zidovudine (**6**), coupling reagents, and DCC and DMAP as catalysts. The mixture was stirred at 0 °C for 15 min and left overnight at room temperature. After workup and purification, the final succinylated oxadiazole-zidovudine (**21**) hybrid was obtained.

(3-Azido-tetrahydro-5-(3,4-dihydro-5-methyl-2,4-dioxopyrimidin-1(2H)-yl)furan-2-yl)methyl 3-(5-(4-chlorophenyl)-1,3,4-oxadiazol-2-ylcarbamoyl)propanoate (**21**): 3-(5-(4-chlorophenyl)-1,3,4-oxadiazol-2-ylcarbamoyl)propanoic acid (**20**) (152 mg, 0.475 mmol, 1.0 equivalent), zidovudine (**6**) (127.0 mg, 0.475 mmol, 1.0 equivalent), DCC (107 mg, 0.522 mmol, 1.1 equivalents), DMAP (5.8 mg, 0.0475 mmol, 0.1 equivalent). Column eluent: DCM/EtOAc (15:3). Yield: 51%. R_f_ = 0.53.

^13^C-NMR (500 MHz, recorded in DMSO-d_6_): chemical shifts (δ, ppm)—178.67 (C-14), 169.28 (C-17), 155.60 (C-10), 154.44 (C-8), 151.02 (C-28), 138.67 (C-32), 134.42 (C-2), 130.36 (C-3), 129.59 (C-1), 128.36 (C-5), 125.06 (C-4), 107.85 (C-31), 77.34 (C-25), 55.28 (C-22), 52.89 (C-21), 47.52 (C-15), 47.32 (C-16), 30.67 (C2-4), 27.48 (C-23), 15.80 (C-33) (Figure S9).

^1^H-NMR (500 MHz, recorded in DMSO-d_6_): chemical shifts (δ, ppm)—8.67 (H-29, singlet, 1H), 8.33 (H-13, singlet, 1H), 7.80 (H-32, singlet, 1H), 7.53 (H-6, doublet, 1H, *J* = 10.0 Hz), 7.51 (H-4, doublet, 1H, *J* = 10.0 Hz), 7.43 (H-1, doublet, 1H, *J* = 10.0 Hz), 7.43 (H-3, doublet, 1H, *J* = 10.0 Hz), 6.73–6.69 (H-24, doublet of a doublet, 2H), 6.57 (H-21, doublet, 2H, *J* = 10.0 Hz), 5.98–5.90 (H-22, multiplet, 1H), 5.08–5.01 (H-24, multiplet, 1H), 3.32 (H-33, singlet, 3H), 3.27 (H-25, doublet, 1H, *J* = 10.0 Hz), 2.72 (H-16, triplet, 2H, *J* = 5.0 Hz), 2.63 (H-15, triplet, 2H, *J* = 5.0 Hz) (Figure S9).

FT–IR (KBr, ν in cm^−1^): 3535–3393 (NH stretching), 2932–2855 (C–H stretching from alkanes). 1704–1626 (C=O stretching), 1494–1452 (C=C stretching (aromatic or alkene)), 1379–1253 (C–H bending (alkanes or aromatics)), 1038–500 (C–O stretching, C–Cl bonds, or skeletal vibrations from aromatic rings) (Figure S19).

HRMS (ESI) mass spectrometry analysis (*m*/*z*) of [C_22_H_21_ClN_8_O_7_]^+^: calculated 544.1222, found 544.1254 (Figure S29).

#### 3.2.5. Synthetic Procedure for Hybrid Compound **23**

Compound **23** was synthesized by following a modified procedure based on the method reported by Hou et al. [54]. Eugenol (**1**) (500 mg, 3.056 mmol, 1.0 equiv.) was first esterified with succinic anhydride (365.9 mg, 3.655 mmol, 1.2 equiv.) in anhydrous DMF (15 mL) using a catalytic amount of DMAP (55.9 mg, 0.457 mmol, 0.15 equiv.). The reaction mixture was stirred at room temperature under an inert atmosphere overnight. The progress of the reaction was monitored by TLC, and the product was purified by column chromatography. The obtained eugenol–succinic acid intermediate (**22**) was then dissolved in anhydrous DMF, and DCC and TEA were added. The mixture was stirred in an ice bath for 15 min before adding fluconazole (**5**), followed by overnight stirring at room temperature. After completion, the reaction mixture was filtered to remove by-products, and the crude product was extracted with ethyl acetate, washed with brine and water, dried over Na_2_SO_4_, and purified by column chromatography.

4-Allyl-2-methoxyphenyl 2-(3,4-difluorophenyl)-1,3-di(1H-1,2,4-triazol-1-yl)propan-2-yl succinate(**23**): fluconazole (150 mg, 0.4900 mmol, 1.0 equivalent), 3-(4-allyl-2-methoxyphenoxy)carbonyl)propanoic acid (**22**) (129.05 mg, 0.4900 mmol, 1.00 equivalent), DCC (101.0 mg, 0.4900 mmol, 1.00 equivalent), TEA (49.6 mg, 0.4900 mmol, 1.00 equivalent), DMF (10 mL). Column eluent: EtOAc/Hex (6:10). Yield: 53%. R_f_ = 0.67.

^13^C-NMR (500 MHz, recorded in DMSO-d_6_): chemical shifts (δ, ppm)—172.86 (C-15), 170.54 (C-11), 154.59 (C-37), 154.44 (C-32), 152.74 (C-6), 151.27 (C-5), 145.58 (C-23), 145.58 (C-27), 135.70 (C-35), 131.66 (C-34), 130.28 (C-21), 126.08 (C-18), 123.94 (C-27), 120.09 (C-2), 120.09 (C-19), 116.05 (C-20), 111.42 (C-4), 111.28 (C-28), 104.60 (C-1), 104.39 (C-22), 74.15 (C-7), 55.42 (C-29), 29.93 (C-8), 26.50 (C-26), 23.06 (C-14), 21.10 (C-13) (Figure S10).

^1^H-NMR (500 MHz, recorded in DMSO-d_6_): chemical shifts (δ, ppm)—8.33 (H-32, singlet, 1H), 8.33 (H-37, singlet, 1H), 7.80 (H-34, singlet, 1H), 7.80 (H-35, singlet, 1H), 6.73 (H-4, singlet, 1H), 6.70 (H-2, doublet, 1H, *J* = 5.0 Hz), 6.70 (H-19, doublet, 1H, *J* = 5.0 Hz), 6.58 (H-22, singlet, 1H), 6.57 (H-1, doublet, 1H, *J* = 10.0 Hz), 6.57 (H-20, doublet, 1H, *J* = 10.0 Hz,), 5.98–5.90 (H-27, multiplet, 1H), 5.04–5.03 (H-28, doublet, 2H, *J* = 5.0 Hz), 3.75 (H-8, singlet, 2H), 3.75 (H-29, singlet, 2H), 3.32 (H-25, singlet, 3H), 3.26 (H-26, doublet, 1H, *J* = 10.0 Hz), 2.89 (H-14, triplet, 2H, *J* = 5.0 Hz), 2.73 (H-13, triplet, 2H, *J* = 5.0 Hz) (Figure S10).

FT–IR (KBr, ν in cm^−1^): 2945–2905 (C–H stretching from alkanes). 1710 (C=O stretching), 1471 (C=C stretching (aromatic or alkene)), 1431–1258 (C–H bending (alkanes or aromatics)), 1180–500 (C–O stretching, C–Cl bonds, or skeletal vibrations from aromatic rings) (Figure S20).

HRMS (ESI) mass spectrometry analysis (*m*/*z*) of [C_27_H_26_F_2_N_6_O_5_]^+^: calculated 552.1933, observed 552.1919 (Figure S30).

### 3.3. Biological Evaluation

#### Antibacterial Activity

The minimum inhibitory concentration (MIC) determination of the hybrid compounds was conducted as outlined by Fonkui et al. (2018) [55]. Each compound was initially dissolved in DMSO at a concentration of 1 mg/mL. These solutions were serially diluted seven times in 100 µL of nutrient broth within a 96-well plate to achieve final concentrations of 500, 250, 125, 62.5, 31.25, 15.625, and 7.8125 µg/mL. Following this, 100 µL of each solution was plated in duplicate with 100 µL of a 0.5 McFarland bacterial suspension. Streptomycin (STM), ampicillin (AMP), nalidixic acid (NLD), and ciprofloxacin (**8**) were included as positive controls, while a DMSO and nutrient broth solution served as the negative control. The MIC values for all the evaluated hybrid molecules are presented in Table 1.

### 3.4. In Silico Studies

#### SwissADME Web Tool

In recent years, in silico studies have become highly popular in drug discovery and the development of small molecules. The SwissADME web tool allows the prediction of key pharmacokinetics, physicochemical properties, drug-likeness, and related characteristics for one or more molecules [56,57,58]. The free online software was accessed directly from the webpage http://www.swissadme.ch (accessed on 25 March 2025). In this study, the web was utilized to evaluate the physicochemical properties, lipophilicity, water solubility, pharmacokinetic profile, drug-likeness, and medicinal chemistry of hybrid compounds with promising antibacterial activity, such as compounds **12**–**16**, **19**, **21,** and **23**. The 2D structure of the synthesized compound was drawn using ChemDraw Ultra version 8.0. The structure was then imported, and their SMILES notation was entered. A SwissADME drug design analysis was performed, and the results were recorded. The physicochemical properties, lipophilicity, water solubility, pharmacokinetic profile, drug-likeness, and medicinal chemistry of these hybrid molecules are presented in Table 2, Table 3, Table 4, Table 5 and Table 6, respectively.

## 4. Conclusions

This study reports the successful synthesis and characterization of hybrid compounds (**12**–**19**, **21,** and **23**) developed through esterification or amidation reactions. The in vitro antibacterial evaluation revealed varying activity among the compounds, with compound **16** (ciprofloxacin-based) demonstrating the most potent and broad-spectrum efficacy, particularly against pathogens commonly associated with COVID-19 co-infections. Essential oil-derived hybrids (e.g., **12**–**14**) showed moderate activity, while terpenoid-based hybrids were less effective, emphasizing the impact of structural features on antibacterial potency. These findings support the potential of hybridization strategies to enhance the efficacy of existing antibacterial agents.

In silico analyses further indicated favourable pharmacokinetic and drug-likeness profiles for most hybrids, with good GI absorption, oral bioavailability, and a low risk of CYP450 inhibition. These properties highlight the potential of the selected compounds as lead candidates for future drug development. Moving forward, experimental validation of the pharmacokinetic predictions, toxicity profiling, and structural optimization will be essential to advance these compounds toward therapeutic application.

## Figures and Tables

**Table 1:** Antibacterial activity of the synthesized hybrid molecules.

**MIC (µg/mL)**														
**Gram-Positive Bacteria**														
**Bacterial Strains**	**12**	**13**	**14**	**15**	**16**	**17**	**18**	**19**	**21**	**23**	**AMP**	**STM**	**NLD**	**8**
*Bacillus subtilis* (ATCC19659)	7.81	7.81	7.81	7.81	7.81	125	125	7.81	7.81	7.81	26	16	16	7.81
*Enterococcus faecalis* (ATCC13047)	7.81	7.81	7.81	7.81	7.81	15.6	15.6	7.81	7.81	7.81	26	128	>512	7.81
*Staphylococcus epidermidis* (ATCC14990)	125	125	125	250	7.81	125	62.5	125	250	500	26	8	64	31.25
*Staphylococcus aureous* (ATCC25923)	7.81	125	125	250	7.81	125	125	125	125	500	26	256	64	125
*Mycobaterium smegmatis* (MC2155)	250	250	250	250	7.81	125	125	250	250	250	26	4	512	250
**Gram-Negative Bacteria**														
*Enterobacter cloacae* (ATCC13047)	7.81	7.81	7.81	7.81	7.81	125	31.2	7.81	7.81	7.81	26	512	16	7.81
*Proteus vulgaris* (ATCC6380)	7.81	7.81	7.81	7.81	7.81	125	31.2	7.81	7.81	7.81	416	128	128	7.81
*Klebsiella oxytoca* (ATCC8724)	7.81	7.81	7.81	7.81	7.81	7.81	15.6	7.81	7.81	7.81	26	16	8	7.81
*Pseudomonas aeruginosa* (ATCC27853)	7.81	7.81	7.81	7.81	7.81	125	31.2	7.81	7.81	7.81	64	128	128	7.81
*Proteus mirabilis* (ATCC7002)	125	125	125	125	7.81	125	31.2	125	125	125	26	128	32	125
*Escherischia coli* (ATCC25922)	125	250	125	250	7.81	250	250	250	250	250	26	64	512	250
*Klebsiella pneumonia* (ATCC13882)	250	250	250	250	7.81	125	62.5	250	250	250	26	512	256	250

**Table 2:** The physicochemical properties of the synthesized hybrid compounds (**12**–**16**, **19**, **21** and **23**).

Physicochemical Properties										
Hybrids	Formula	Molecular Weight	Num. of Heavy Atoms	Num. of Arom. Heavy Atoms	Fraction Csp3	Num. of Rotatable Bonds	Num. of H-Bond Acceptors	Num. of H-Bond Donors	Molar Refractivity	TPSA
**12**	C_20_H_18_ClN_3_O_4_	399.83 g/mol	28	17	0.15	9	6	1	105.67	86.48 Å^2^
**13**	C_20_H_20_ClN_3_O_3_	385.84 g/mol	27	17	0.25	7	5	1	104.62	77.25 Å^2^
**14**	C_20_H_20_ClN_3_O_3_	385.84 g/mol	27	17	0.25	7	5	1	104.62	77.25 Å^2^
**15**	C_18_H_12_Cl_2_N_6_O_3_	431.23 g/mol	29	22	0.06	7	7	2	106.19	118.97 Å^2^
**16**	C_27_H_24_ClFN_6_O_5_	566.97 g/mol	40	21	0.30	8	8	2	152.28	133.80 Å^2^
**19**	C_27_H_32_ClN_3_O_8_	562.01 g/mol	39	11	0.63	8	10	1	137.98	131.24 Å^2^
**21**	C_22_H_21_ClN_8_O_7_	544.90 g/mol	38	17	0.36	11	12	2	128.41	208.16 Å^2^
**23**	C_27_H_26_F_2_N_6_O_5_	552.53 g/mol	40	22	0.26	15	11	0	136.87	123.25 Å^2^

**Table 3:** The characteristics of the lipophilicity of the hybrid compounds (**12**–**16**, **19**, **21** and **23**).

Lipophilicity						
Hybrids	Log P_o/w_ (iLOGP)	Log P_o/w_ (XLOGP3)	Log P_o/w_ (WLOGP)	Log P_o/w_ (MLOGP)	Log P_o/w_ (SILICOS-IT)	Consensus Log P_o/w_
**12**	3.36	4.29	3.95	2.54	4.34	3.70
**13**	3.40	4.74	4.65	3.15	4.63	4.11
**14**	3.45	4.74	4.65	3.15	4.63	4.12
**15**	3.00	3.71	3.76	2.68	3.15	3.26
**16**	3.03	3.29	3.04	2.24	2.84	2.89
**19**	4.43	4.53	4.53	3.43	2.73	3.93
**21**	3.83	1.37	1.96	0.14	1.14	1.69
**23**	3.76	3.45	4.14	3.23	3.87	3.69

**Table 4:** The characteristics of the water solubility of the hybrid compounds (**12**–**16**, **19**, **21** and **23**).

Water Solubility									
Hybrids	Log *S* (ESOL)	Solubility	Class	Log *S* (Ali)	Solubility	Class	Log *S* (SILICOS-IT)	Solubility	Class
**12**	−4.88	5.31 × 10^−3^ mg/mL; 1.33 × 10^−5^ mol/L	Moderately soluble	−5.82	6.07 × 10^−4^ mg/mL; 1.52 × 10^−6^ mol/L	Moderately soluble	−7.656	1.09 × 10^−5^ mg/mL; 2.74 × 10^−8^ mol/L	Poorly soluble
**13**	−5.22	2.31 × 10^−3^ mg/mL; 5.99 × 10^−6^ mol/L	Moderately soluble	−6.09	3.12 × 10^−4^ mg/mL; 8.09 × 10^−7^ mol/L	Poorly soluble	−7.81	6.03 × 10^−6^ mg/mL; 1.56 × 10^−8^ mol/L	Poorly soluble
**14**	−6.09	2.31 × 10^−3^ mg/mL; 5.99 × 10^−6^ mol/L	Moderately soluble	−6.09	3.12 × 10^−4^ mg/mL; 8.09 × 10^−7^ mol/L	Poorly soluble	−7.81	6.03 × 10^−6^ mg/mL; 1.56 × 10^−8^ mol/L	Poorly soluble
**15**	−4.95	4.83 × 10^−3^ mg/mL; 1.12 × 10^−5^ mol/L	Moderately soluble	−5.90	5.44 × 10^−4^ mg/mL; 1.26 × 10^−6^ mol/L	Moderately soluble	−8.47	1.46 × 10^−6^ mg/mL; 3.40 × 10^−9^ mol/L	Poorly soluble
**16**	−5.29	2.92 × 10^−3^ mg/mL; 5.15 × 10^−6^ mol/L	Moderately soluble	−5.78	9.52 × 10^−4^ mg/mL; 1.68 × 10^−6^ mol/L	Moderately soluble	−7.08	4.69 × 10^−5^ mg/mL; 8.28 × 10^−8^ mol/L	Poorly soluble
**19**	−5.86	7.77 × 10^−4^ mg/mL; 1.38 × 10^−6^ mol/L	Poorly soluble	−7.01	5.52 × 10^−5^ mg/mL; 9.82 × 10^−8^ mol/L	Poorly soluble	−5.83	8.24 × 10^−4^ mg/mL; 1.47 × 10^−6^ mol/L	Moderately soluble
**21**	−3.69	1.12 × 10^−1^ mg/mL; 2.06 × 10^−4^ mol/L	Soluble	−5.34	2.47 × 10^−3^ mg/mL; 4.53 × 10^−6^ mol/L	Moderately soluble	−6.12	4.11 × 10^−4^ mg/mL; 7.55 × 10^−7^ mol/L	Poorly soluble
**23**	−4.86	7.69 × 10^−3^ mg/mL; 1.39 × 10^−5^ mol/L	Moderately soluble	−5.72	1.05 × 10^−3^ mg/mL; 1.91 × 10^−6^ mol/L	Moderately soluble	−7.57	1.48 × 10^−5^ mg/mL; 2.69 × 10^−8^ mol/L	Poorly soluble

**Table 5:** The pharmacokinetic properties of the synthesized hybrid compounds calculated with the SwissADME database.

Pharmacokinetics									
Hybrids	GI Absorption	BBB Permeant	P-gp Substrate	CYP1A2 Inhibitor	CYP2C19 Inhibitor	CYP2C9 Inhibitor	CYP2D6 Inhibitor	CYP3A4 Inhibitor	Log *K*_p_ (Skin Permeation)
**12**	High	No	No	Yes	Yes	Yes	No	Yes	−5.69 cm/s
**13**	High	No	No	Yes	Yes	Yes	No	Yes	−5.29 cm/s
**14**	High	No	No	Yes	Yes	Yes	No	Yes	−5.29 cm/s
**15**	High	No	No	Yes	Yes	Yes	No	Yes	−6.30 cm/s
**16**	High	No	Yes	No	Yes	Yes	Yes	Yes	−7.42 cm/s
**19**	Low	No	Yes	No	No	No	Yes	Yes	−6.51 cm/s
**21**	Low	No	Yes	No	No	No	No	Yes	−8.65 cm/s
**23**	High	No	No	Yes	Yes	Yes	No	Yes	−7.22 cm/s

**Table 6:** Drug-likeness scores of the synthesized hybrid compounds calculated with the SwissADME database.

Drug-likeness						
Hybrids	Lipinski	Ghose	Veber	Egan	Muegge	Bioavailability Score
**12**	Yes; 0 violations	Yes	Yes	Yes	Yes	0.55
**13**	Yes; 0 violations	Yes	Yes	Yes	Yes	0.55
**14**	Yes; 0 violations	Yes	Yes	Yes	Yes	0.55
**15**	Yes; 0 violations	Yes	Yes	Yes	Yes	0.55
**16**	No; 2 violations: MW > 500, NorO > 10	No; 2 violations: MW > 480, MR > 130	Yes	No; 1 violation: TPSA > 131.6	Yes	0.56
**19**	No; 2 violations: MW > 500, NorO > 10	No; 3 violations: MW > 480, MR > 130, #atoms > 70	Yes	Yes	Yes	0.17
**21**	No; 2 violations: MW > 500, NorO > 10	No; 1 violation: MW > 480	No; 2 violations: Rotors > 10, TPSA > 140	No; 1 violation: TPSA > 131.6	No; 2 violations: TPSA > 150, H-acc > 10	0.17
**23**	No; 2 violations: MW > 500, NorO > 10	No; 2 violations: MW > 480, MR > 130	No; 1 violation: Rotors > 10	Yes	No; 1 violation: H-acc > 10	0.17

**Table 7:** Medicinal chemistry of the synthesized hybrid compounds calculated with the SwissADME database.

Medicinal Chemistry				
Hybrids	PAINS	Brenk	Leadlikeness	Synthetic Accessibility
**12**	0 alerts	1 alert: isolated_alkene	No; 3 violations: MW > 350, Rotors > 7, XLOGP3 > 3.5	3.28
**13**	0 alerts	0 alerts	No; 2 violations: MW > 350, XLOGP3 > 3.5	3.29
**14**	0 alerts	0 alerts	No; 2 violations: MW > 350, XLOGP3 > 3.5	3.29
**15**	0 alerts	0 alerts	No; 2 violations: MW > 350, XLOGP3 > 3.5	3.12
**16**	0 alerts	0 alerts	No; 2 violations: MW > 350, Rotors > 7	3.87
**19**	0 alerts	1 alert: peroxide	No; 3 violations: MW > 350, Rotors > 7, XLOGP3 > 3.5	7.12
**21**	1 alert: azo_A	3 alerts: azido_group, diazo_group, quaternary_nitrogen_3	No; 2 violations: MW > 350, Rotors > 7	5.03
**23**	0 alerts	2 alerts: isolated_alkene, phenol_ester	No; 2 violations: MW > 350, Rotors > 7	3.97

## Data Availability

The original contributions presented in this study are included in the article/Supplementary Materials. Further inquiries can be directed to the corresponding authors.

## Supplementary Materials

The following Supporting Information can be downloaded at https://www.mdpi.com/article/10.3390/antibiotics14060582/s1: original NMR, FTIR, and HRMS spectra of the synthesized compounds **12**–**19**, **21**, and **23**.

## Abbreviations

The following abbreviations are used in this manuscript:

^13^C NMR	Carbon-13 Nuclear Magnetic Resonance
^1^H NMR	Proton Nuclear Magnetic Resonance
AMP	Ampicillin
AMR	Antimicrobial Resistance
CDCl-d_3_	Deuterated Chloroform
DCC	N,N’-Dicyclohexylcarbodiimide
DCM	Dichloromethane
DMAP	4-Dimethylaminopyridine
DMF	Dimethylformamide
DMSO-d_6_	Deuterated Chloroform
DNA	Deoxyribonucleic Acid
EtOAc	Ethyl Acetate
FTIR	Fourier Transform Infrared Spectroscopy
Hex	Hexane
HRMS	High-Resolution Mass Spectrometry
ICU	Intensive Care Unit
MIC	Minimum Inhibitory Concentration
NHS	N-Hydroxysuccinimide
NLD	Nalidixic Acid
STM	Streptomycin
TEA	Triethylamine
TLC	Thin-Layer Chromatography
UV	Ultraviolet
WHO	World Health Organization
