# Supplementary material for: Design and Synthesis of Hybrid Compounds for Potential Treatment of Bacterial Co-Infections: In Vitro Antibacterial and In Silico Studies
Source: Antibiotics (Basel). 2025 Jun 6;14(6):582. doi: 10.3390/antibiotics14060582 (PMC12542292; doi:10.3390/antibiotics14060582)
Supplement: Supplementary file 1 [file antibiotics-14-00582-s001.zip › antibiotics-3580589-supplementary.pdf]

Supplementary Materials

^{1}H and ^{13}C NMR spectra of compounds **12–19, 21** and **23** (Figure S1–Figure S13)

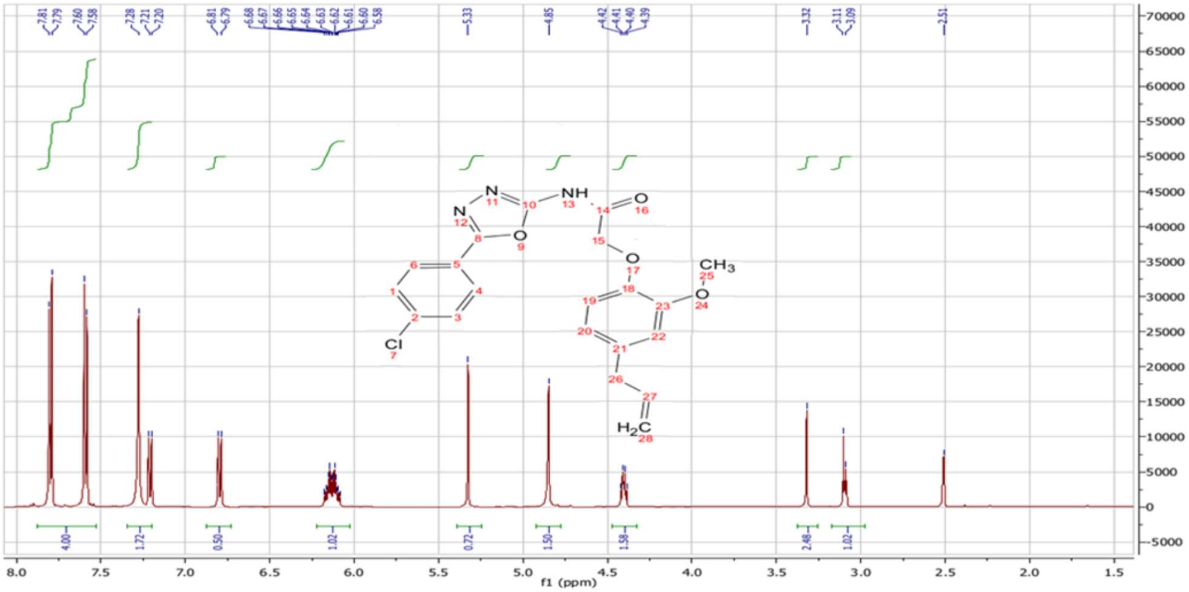

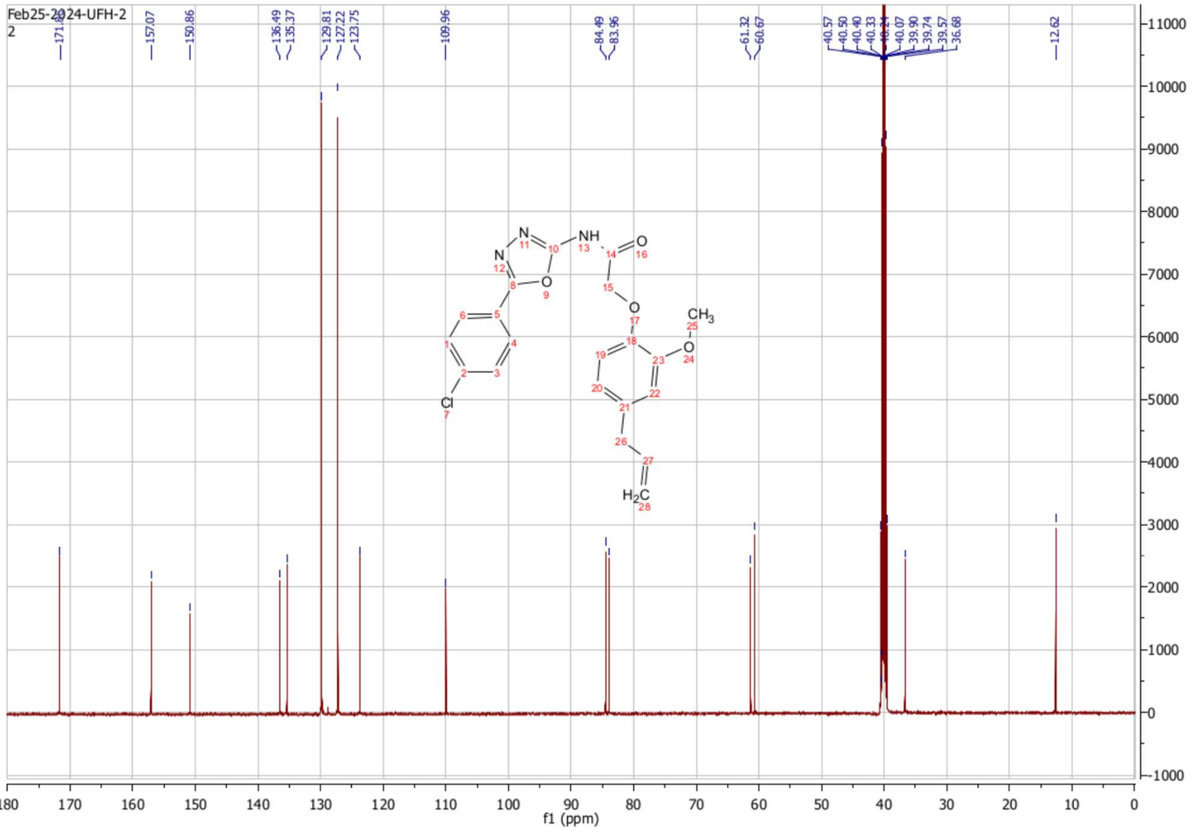

Figure S1: ^{1}H and ^{13}C NMR spectra(DMSO-d_6) of compounds **12**

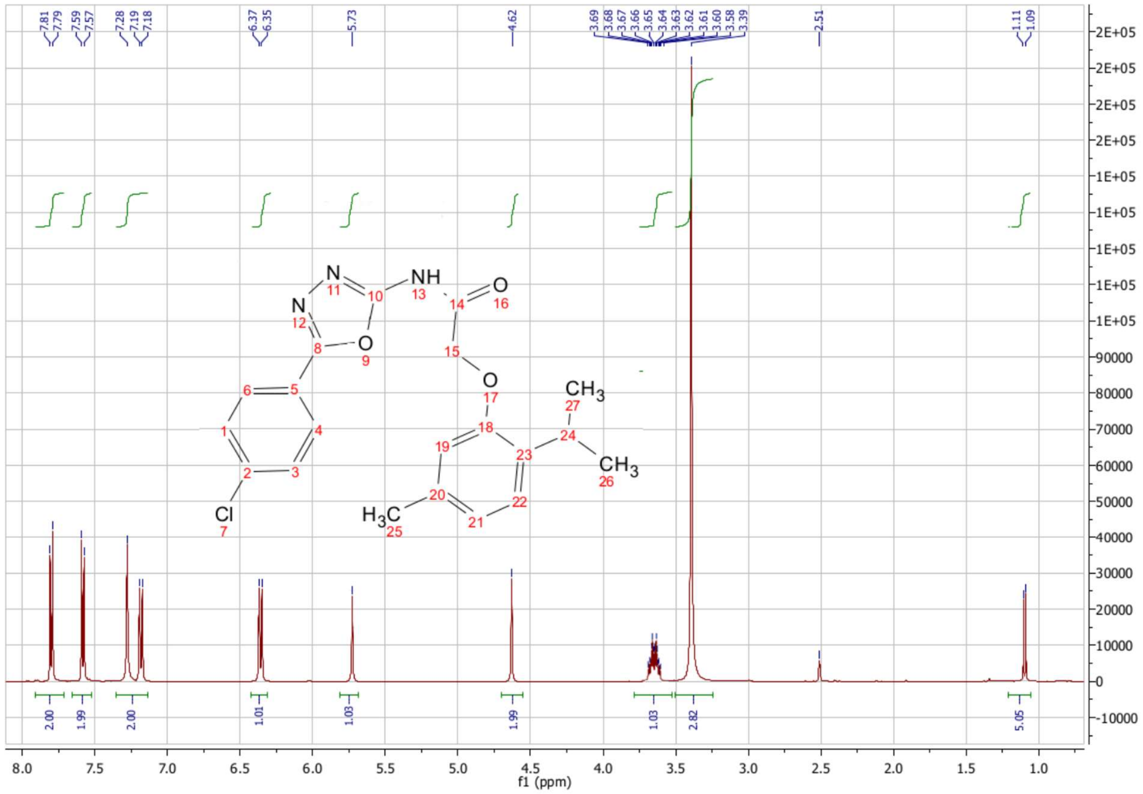

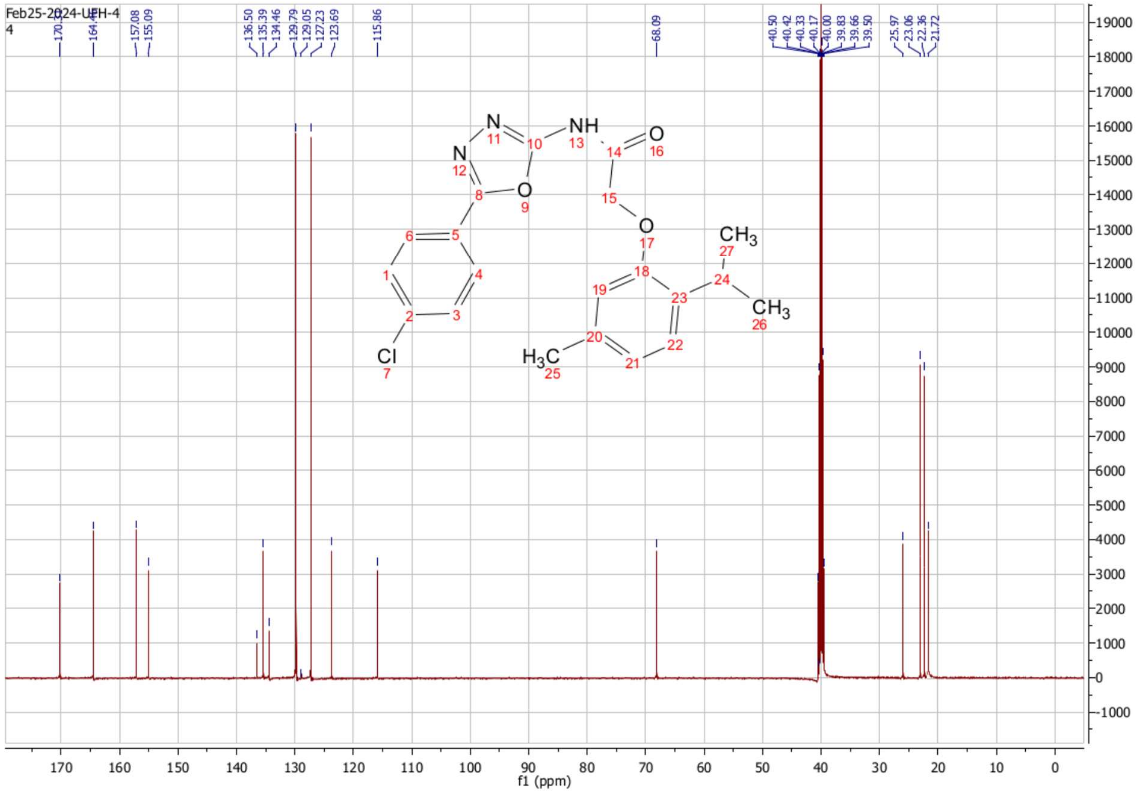

Figure S2: ^{1}H and ^{13}C NMR(recorded DMSO-d_6) spectra of compounds **13**

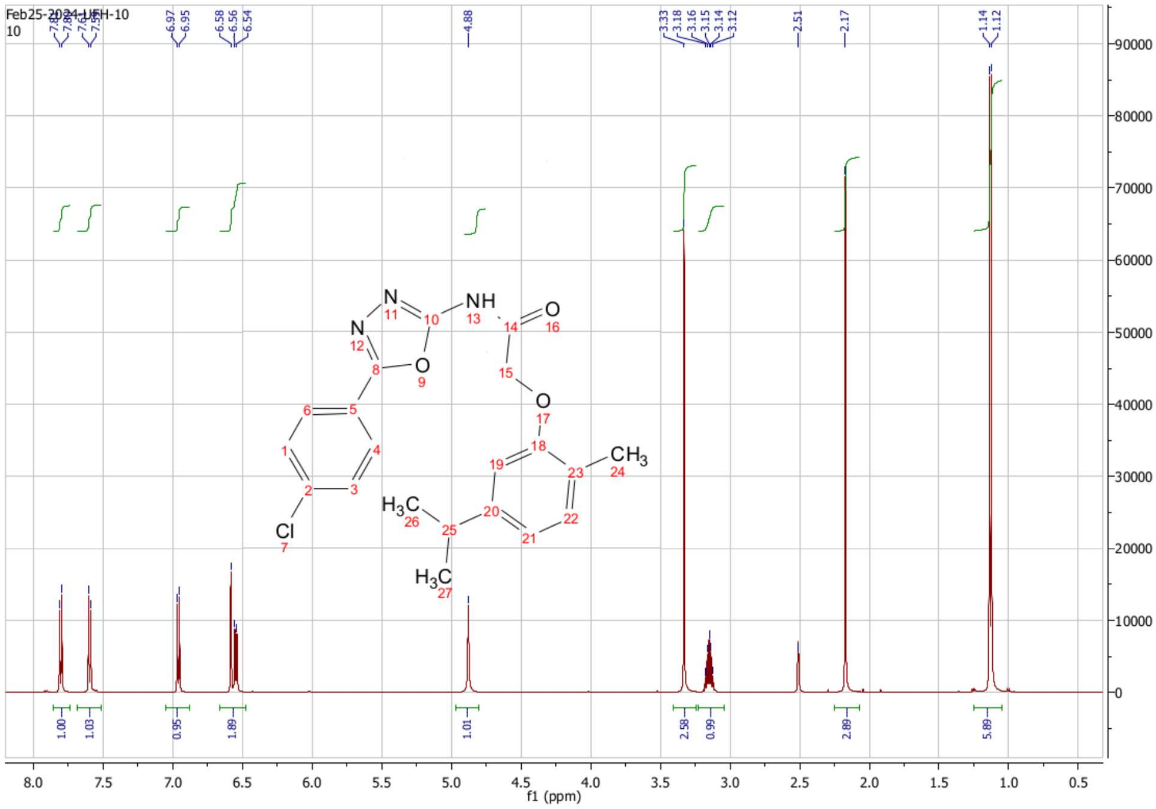

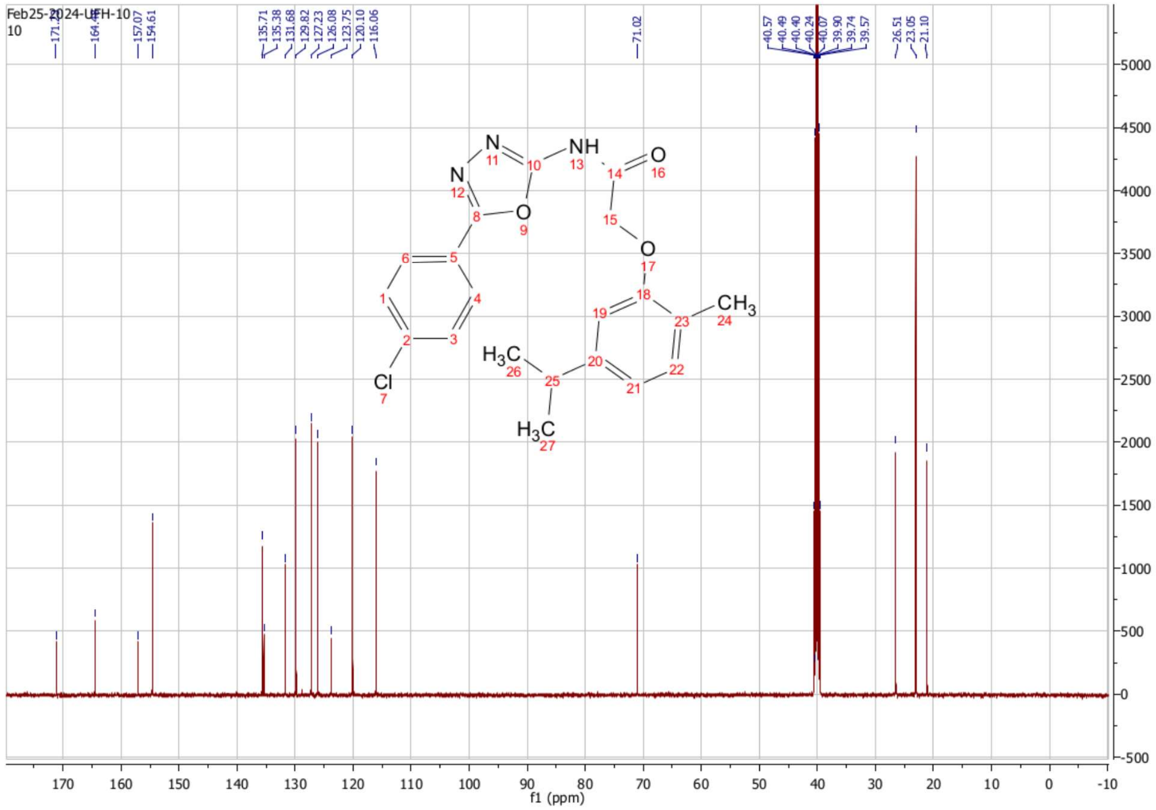

Figure S3: ^{1}H and (recorded DMSO-d6) ^{13}C NMR spectra of compounds **14**

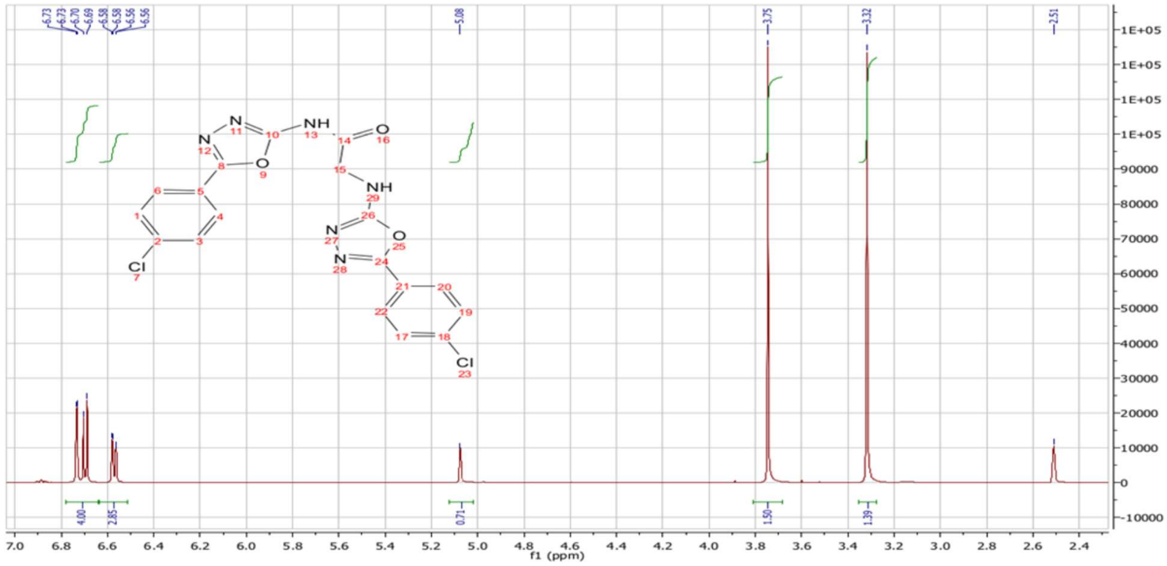

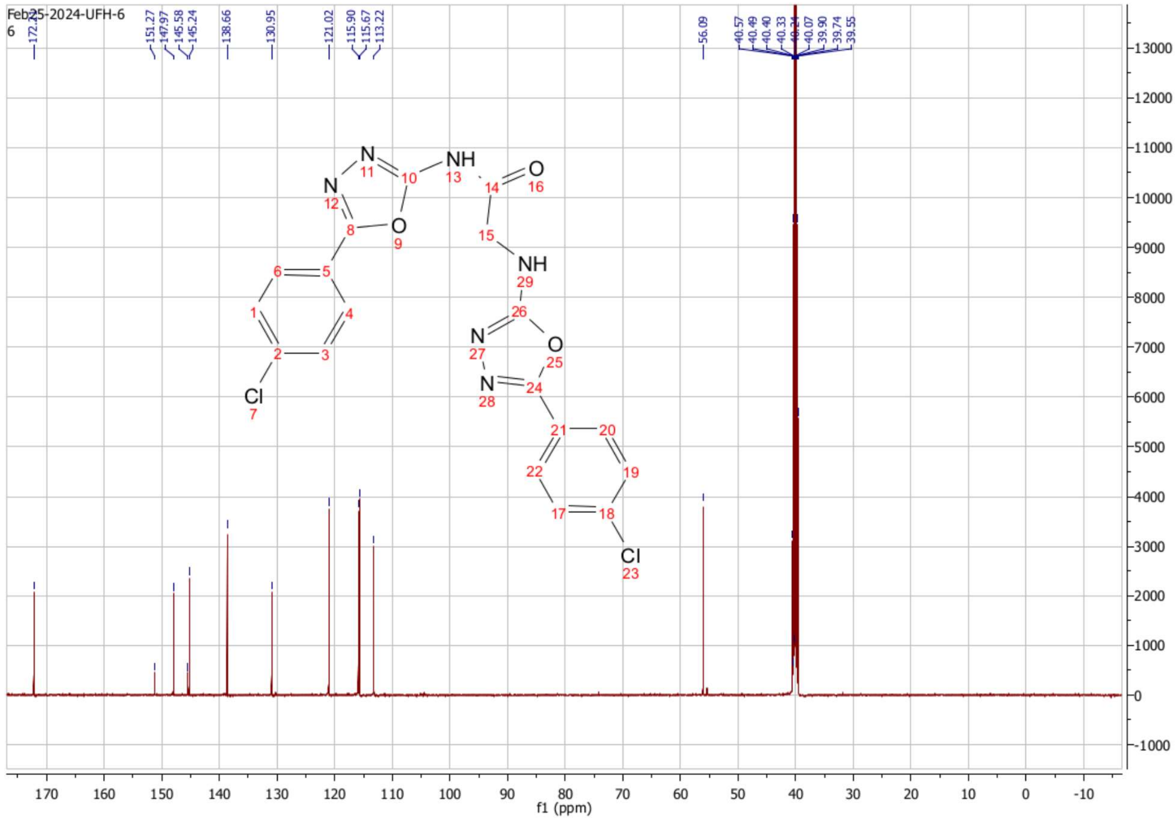

Figure S4: ^{1}H and ^{13}C NMR(recorded DMSO-d_6) spectra of compounds **15**

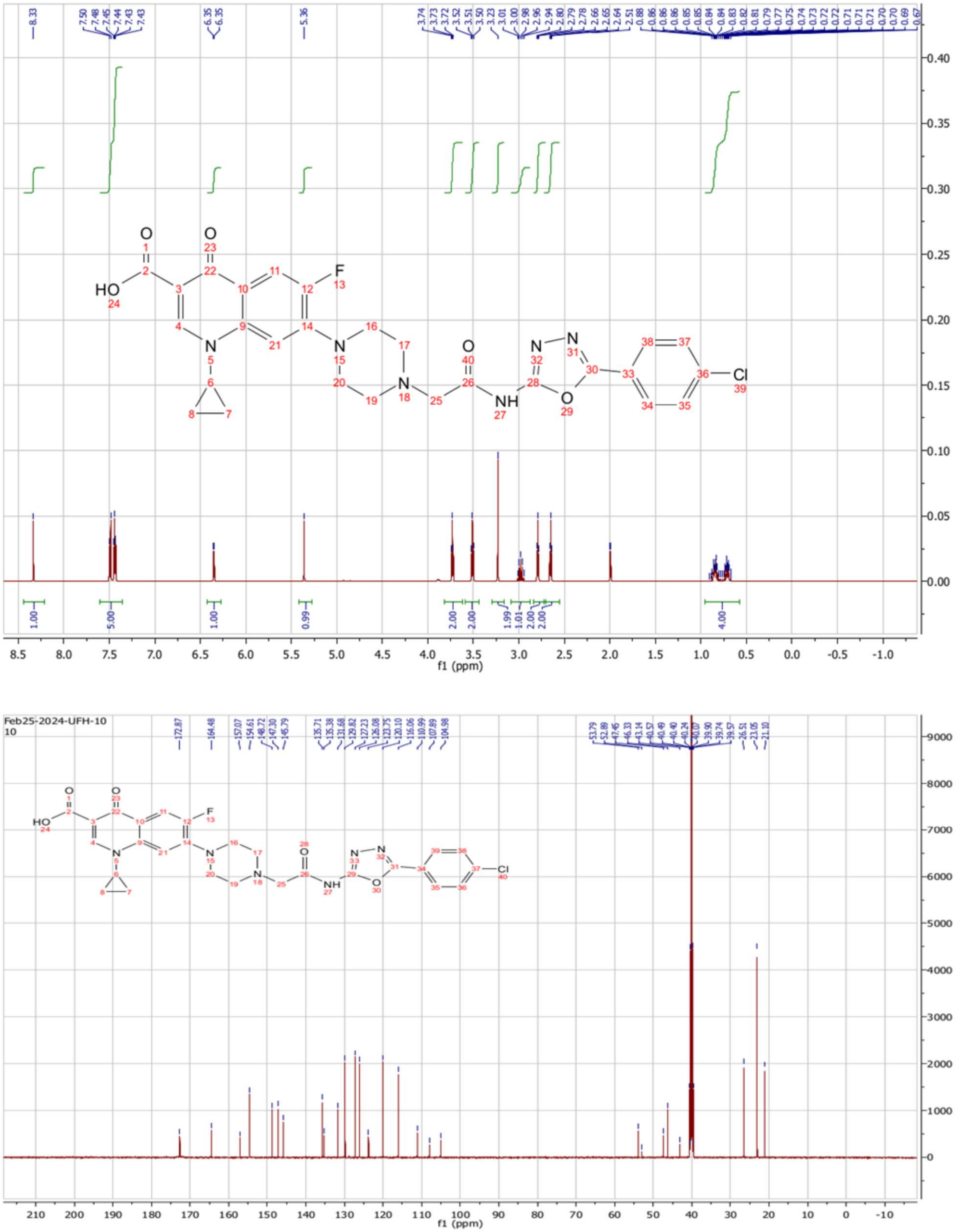

Figure S5: ^{1}H and ^{13}C NMR (recorded DMSO-d$_6$) spectra of compounds **16**

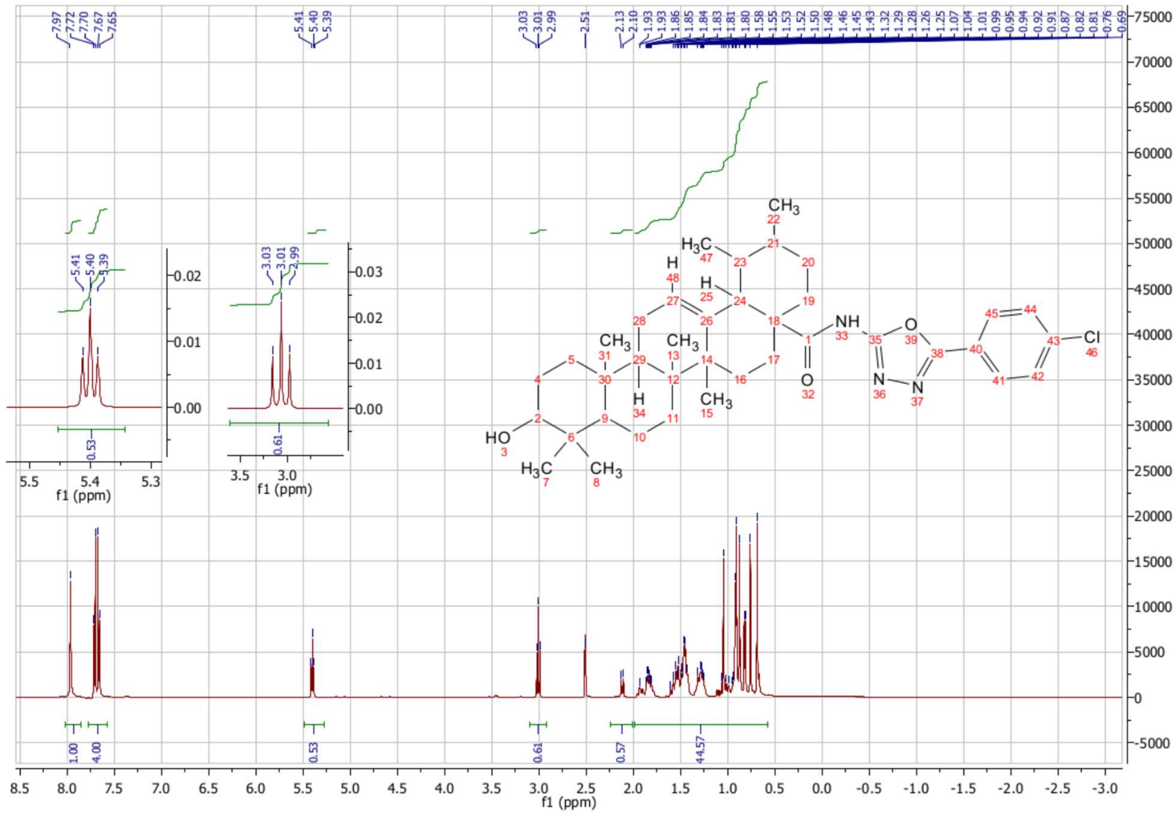

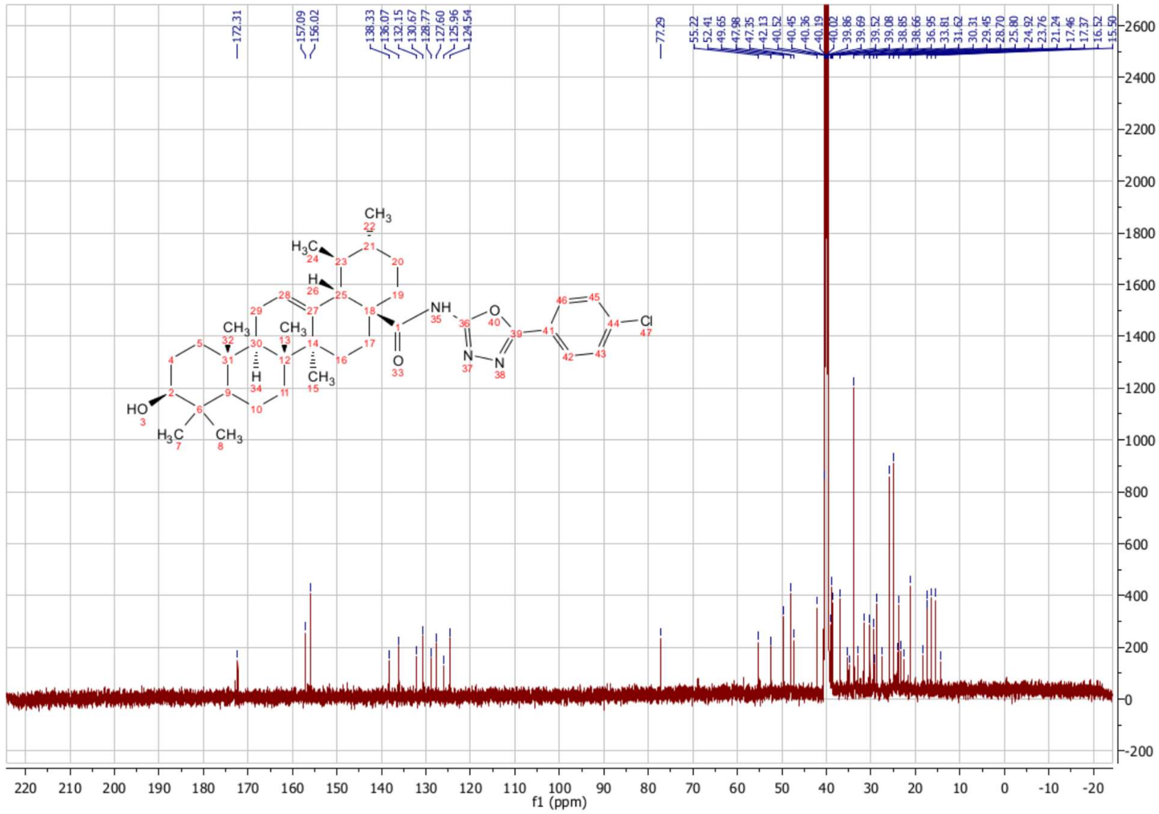

Figure S6: ^{1}H NMR (recorded DMSO-d$_6$) and ^{13}C NMR (recorded in CDCl-d$_3$) spectra of compounds **17**

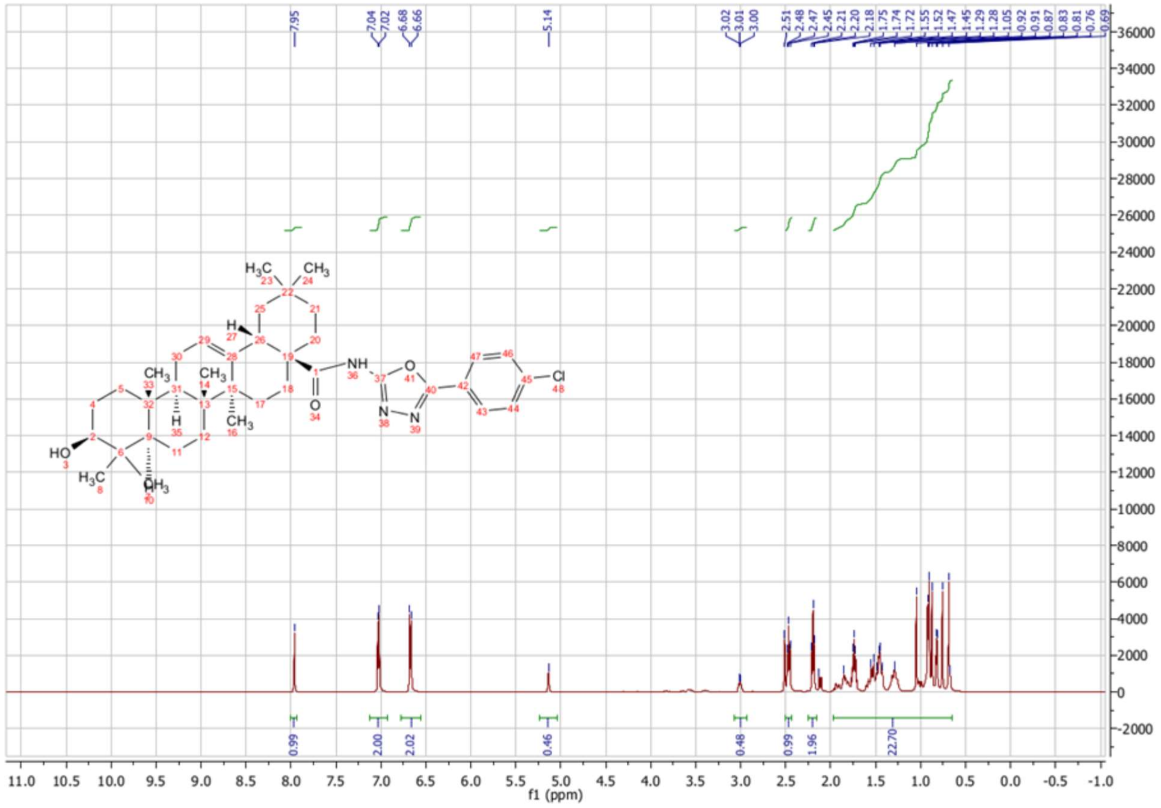

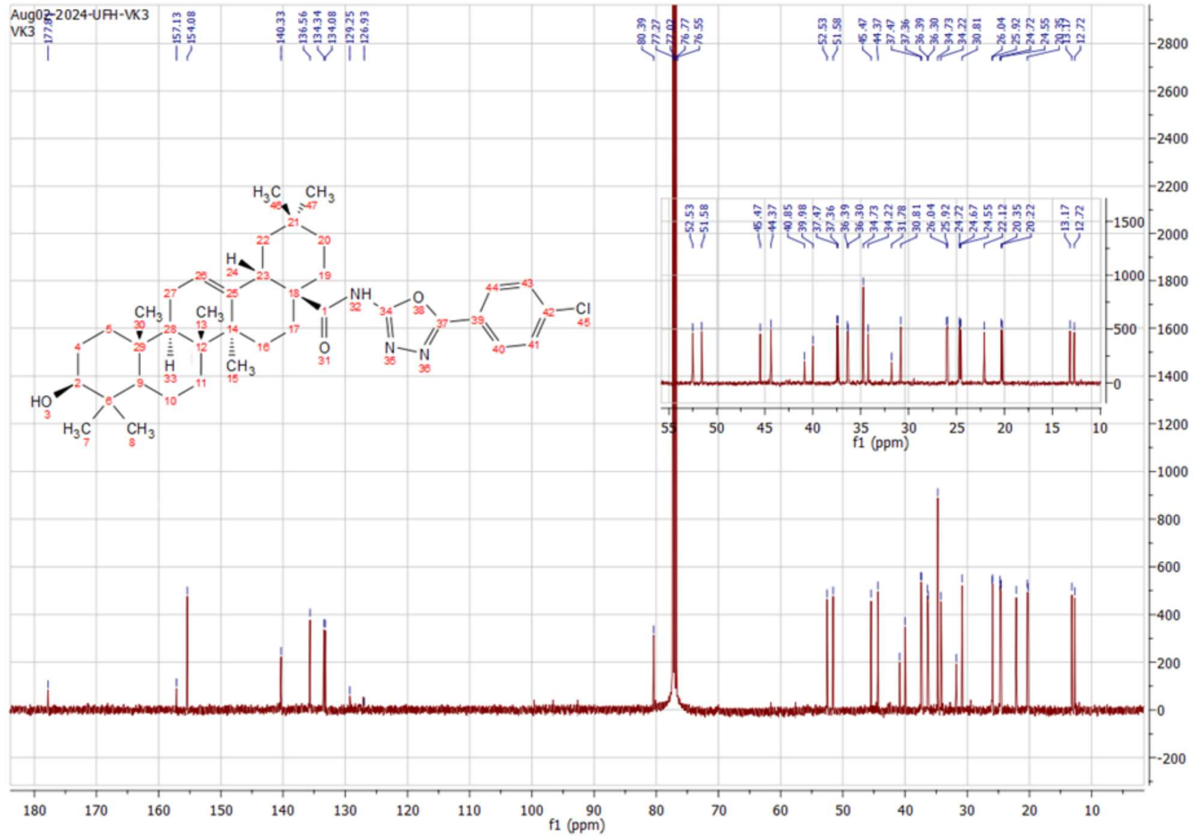

Figure S7: ^{1}H and ^{13}C NMR (recorded DMSO-d6) spectra of compounds **18**

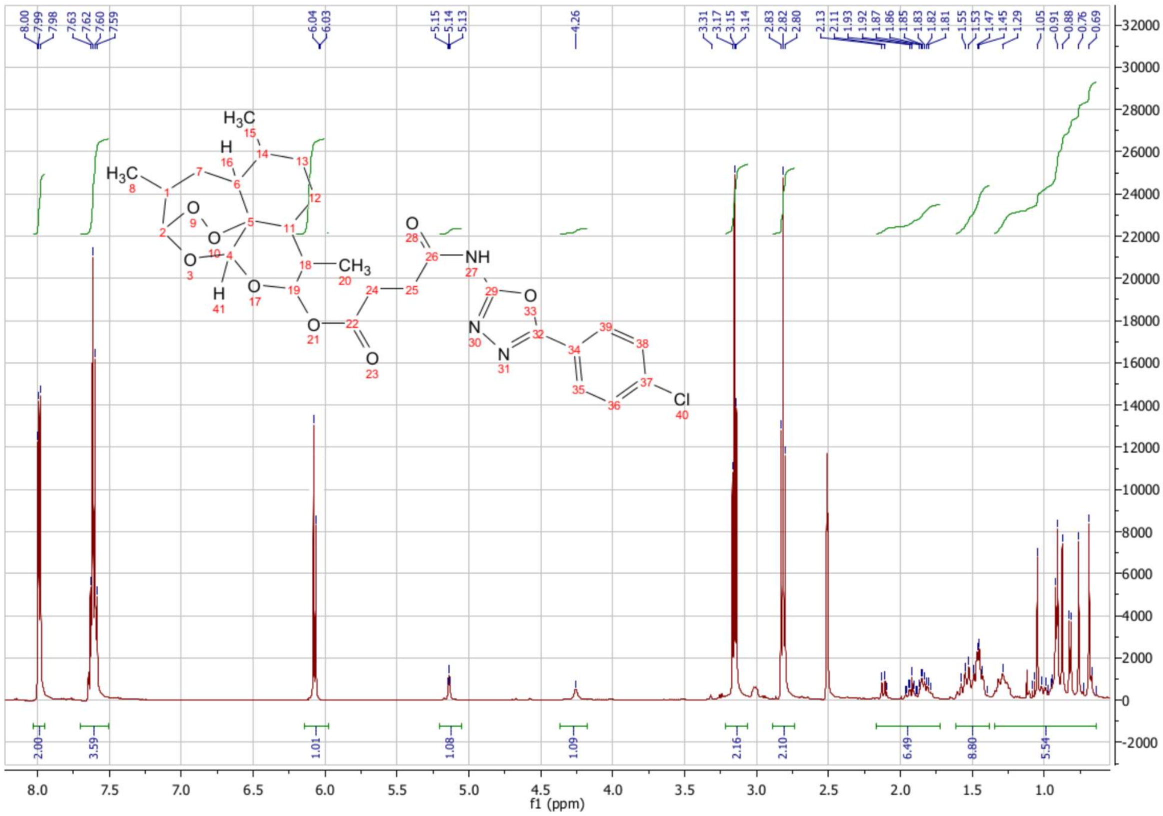

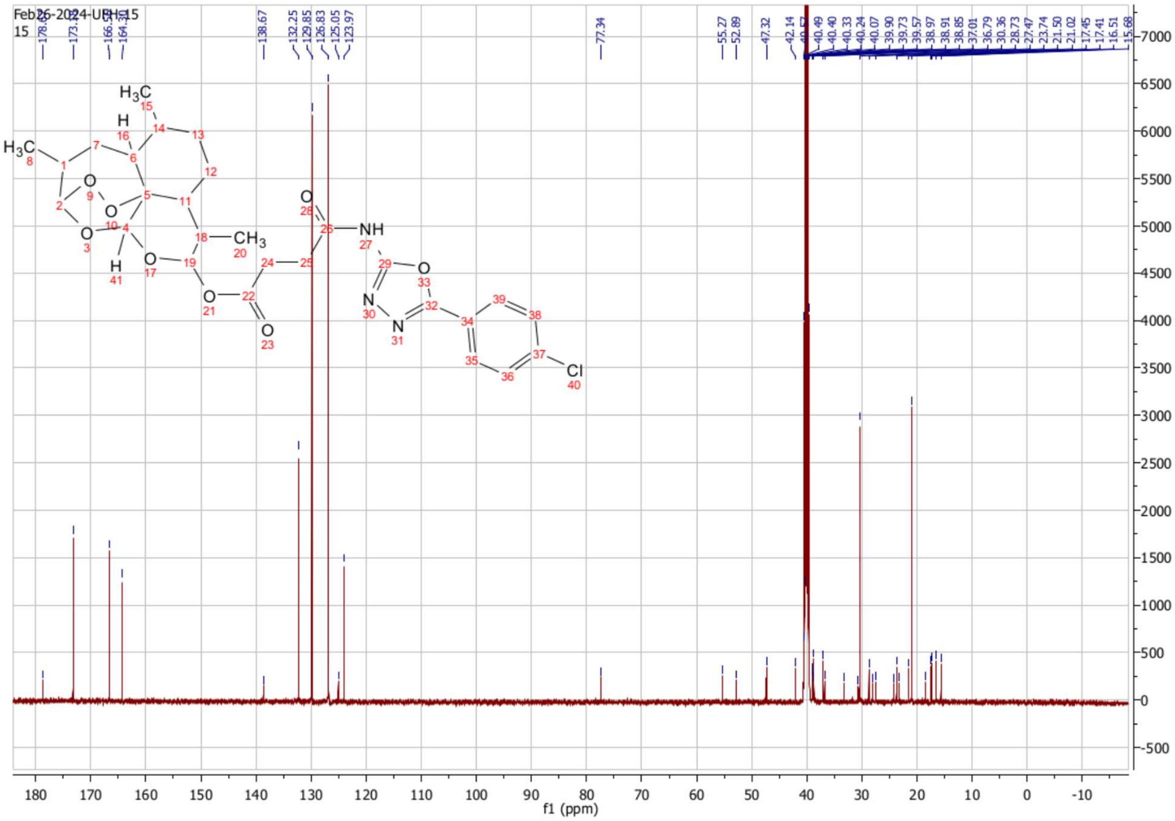

Figure S8: ^{1}H and ^{13}C NMR (recorded DMSO-d_6) spectra of compounds **19**

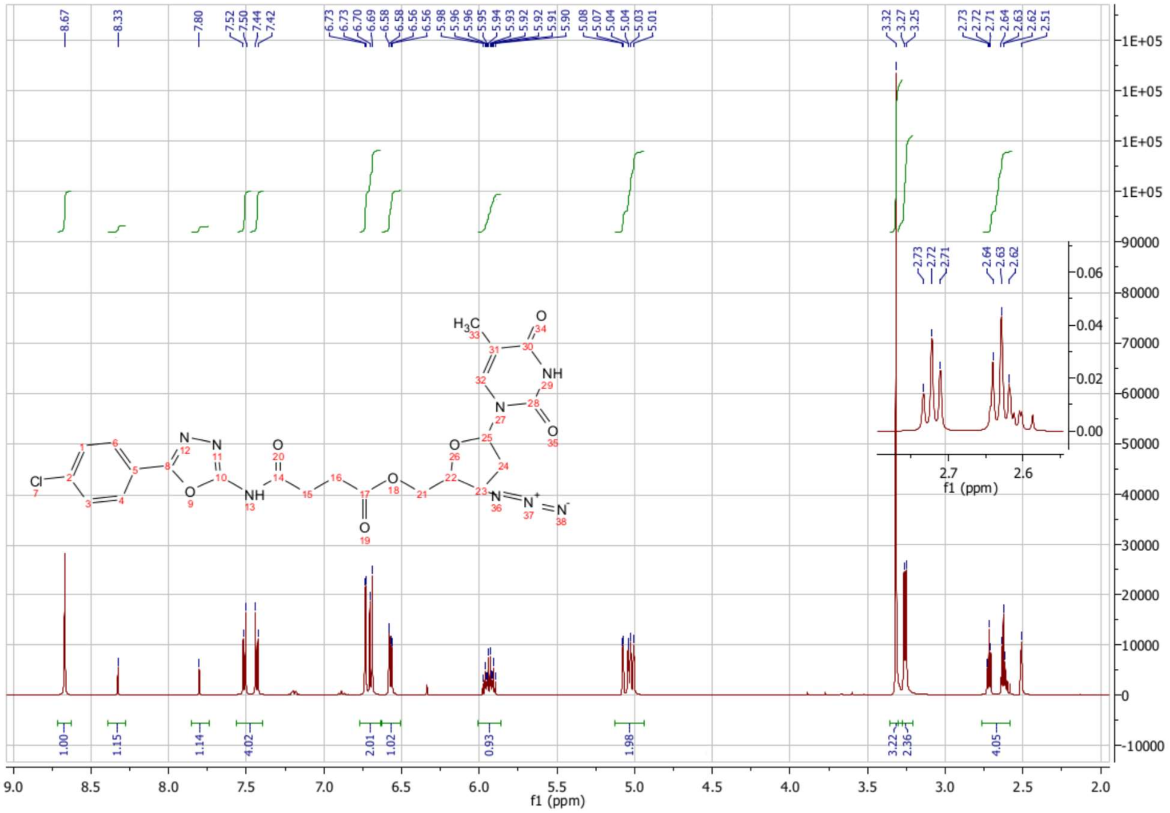

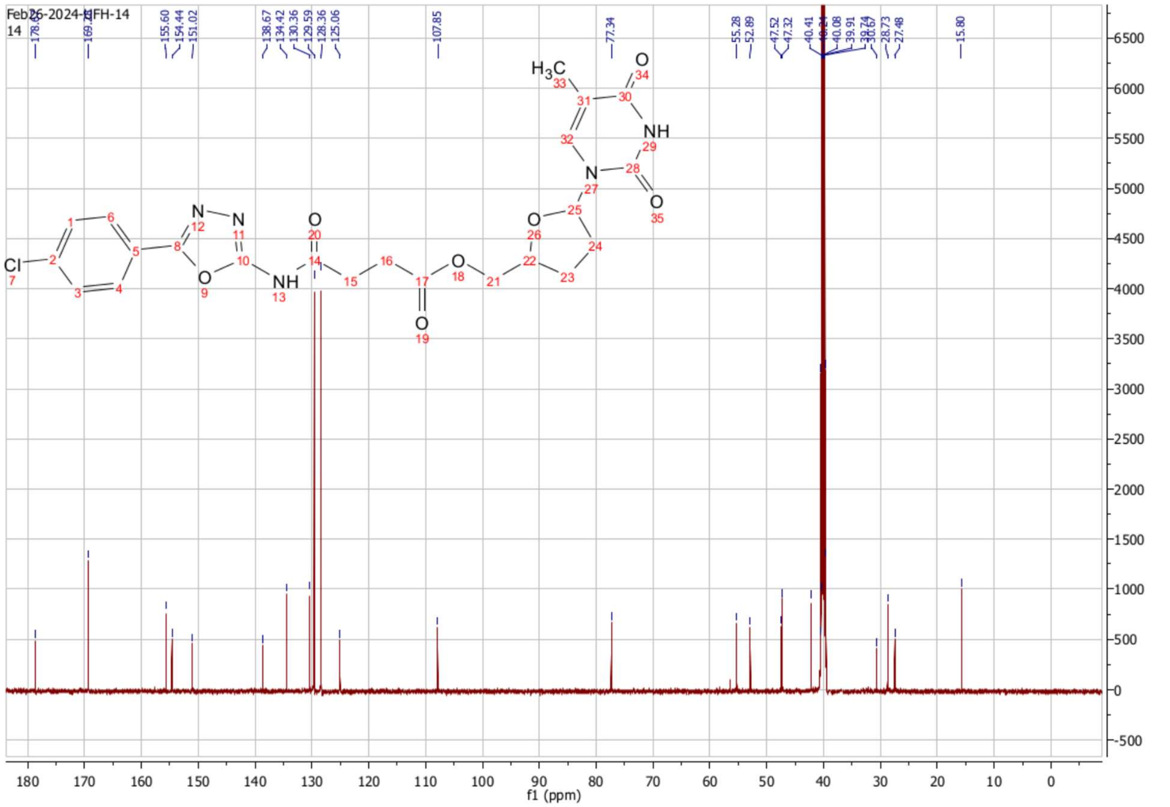

Figure S9: ^{1}H and ^{13}C NMR (recorded DMSO-d_6) spectra of compounds **21**

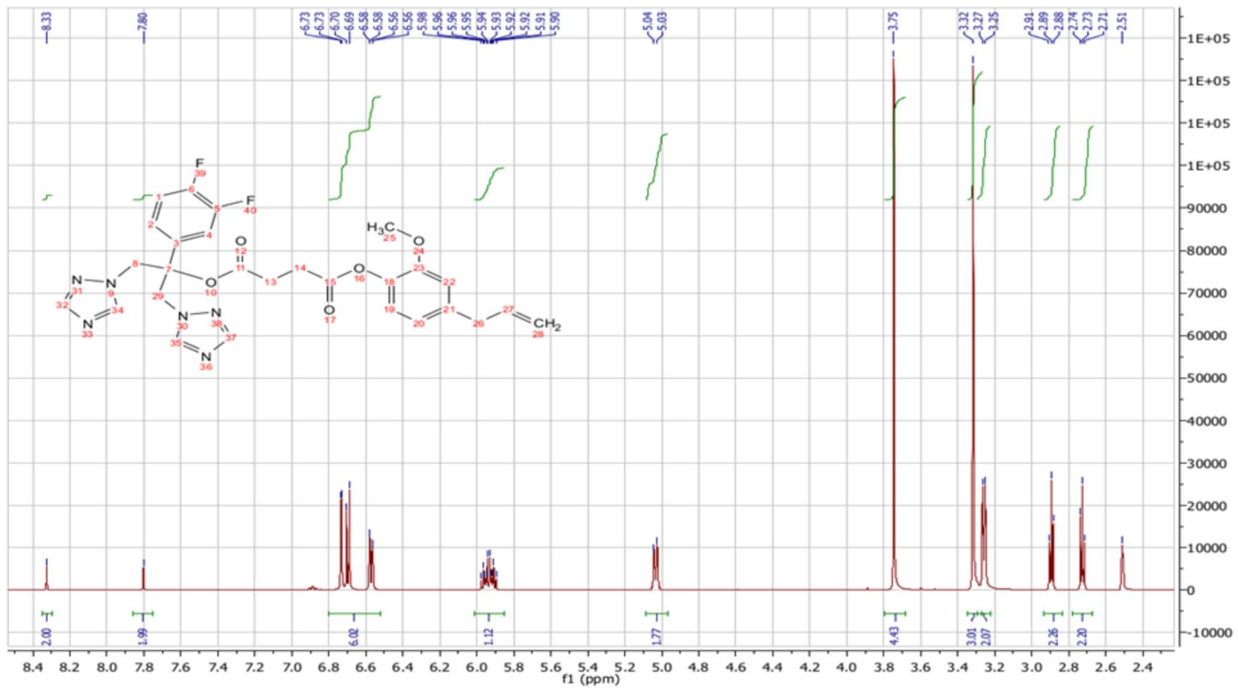

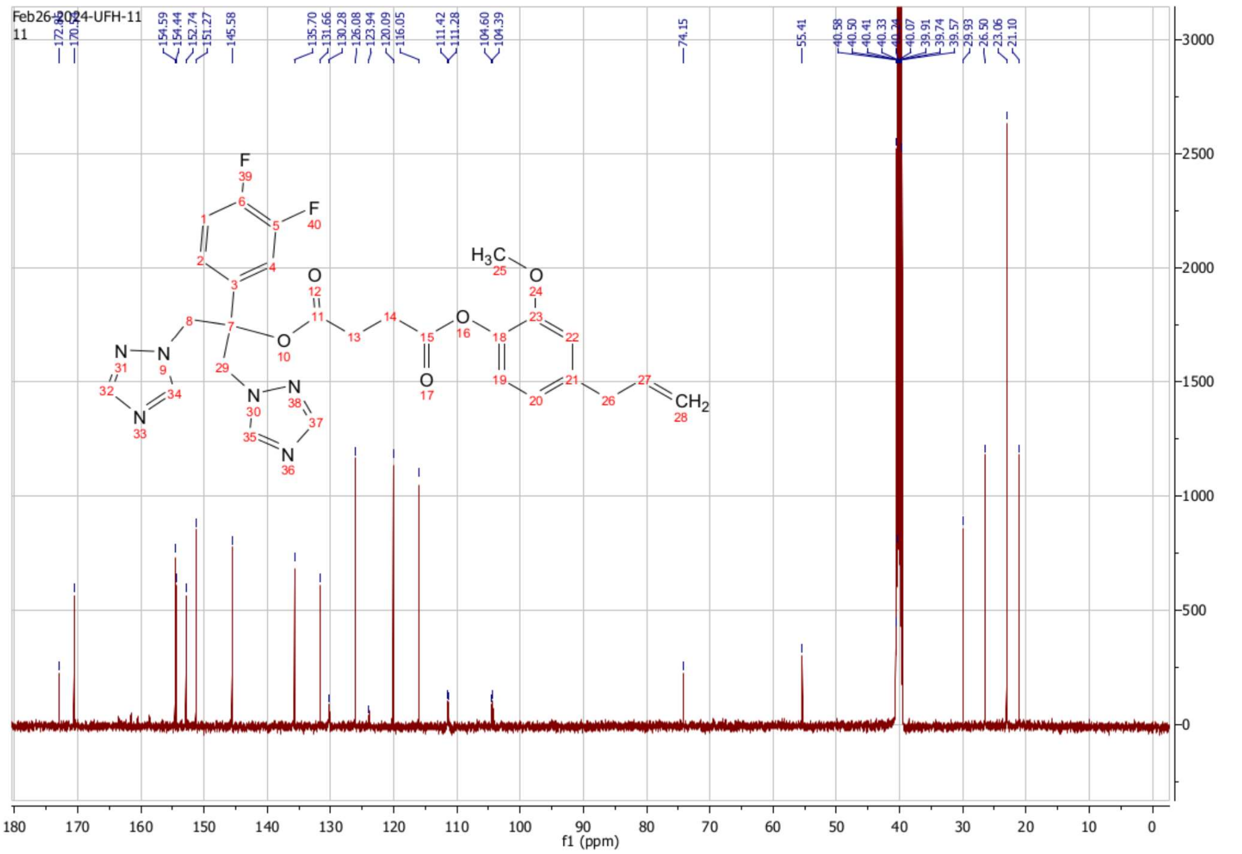

Figure S10: ^{1}H and ^{13}C NMR (recorded DMSO-d_6) spectra of compound **23**

FTIR spectra of compounds **12-19**, **21-23** (Figure S**11**-Figure S**20**)

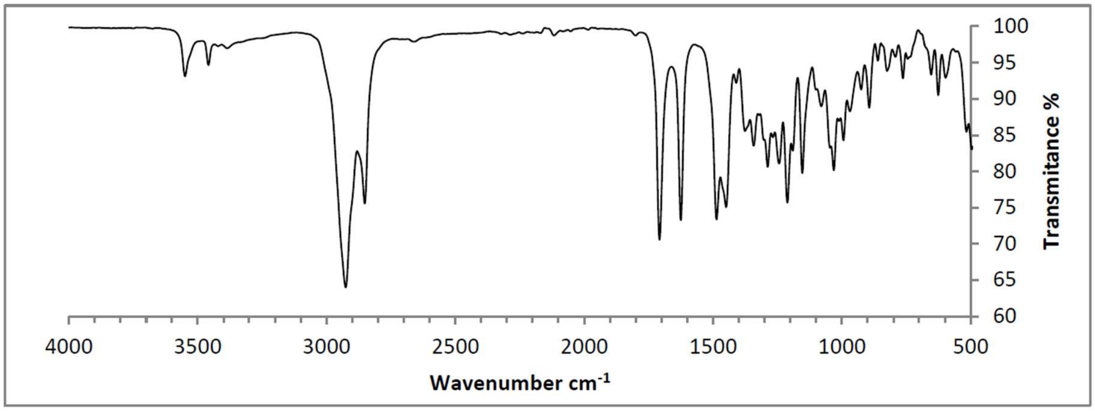

Figure S11: Compound **12**

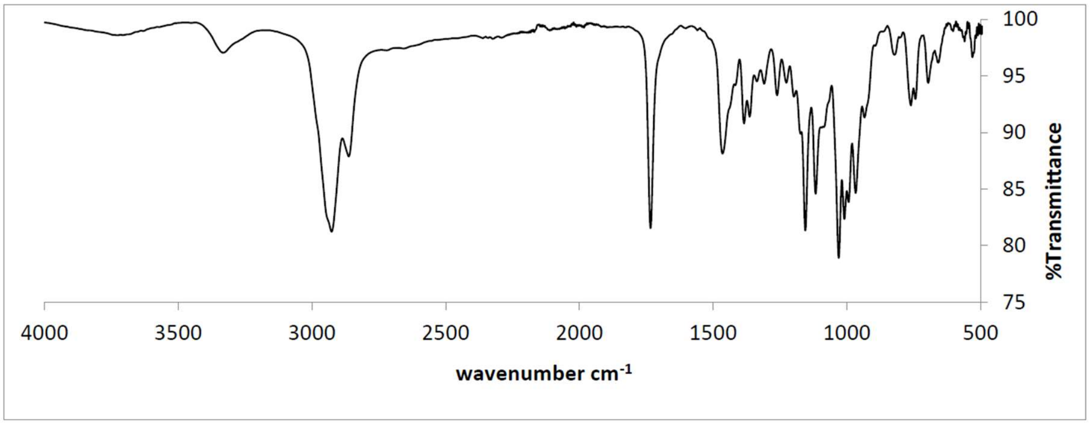

Figure S12: Compound **13**

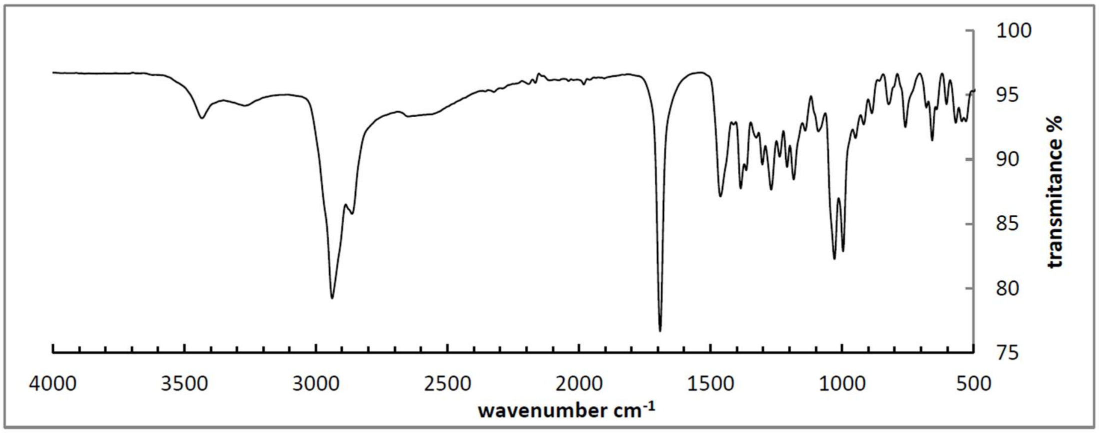

Figure S13: Compound **14**

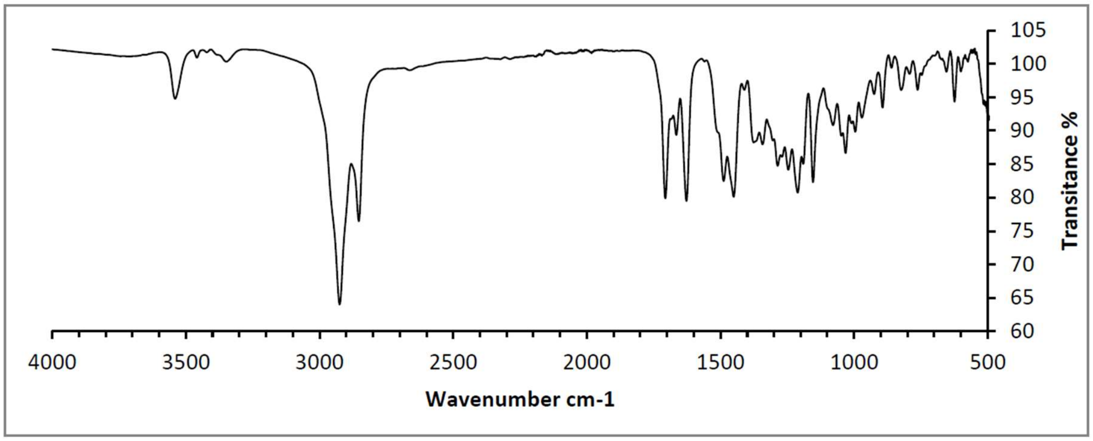

Figure S14: Compound **15**

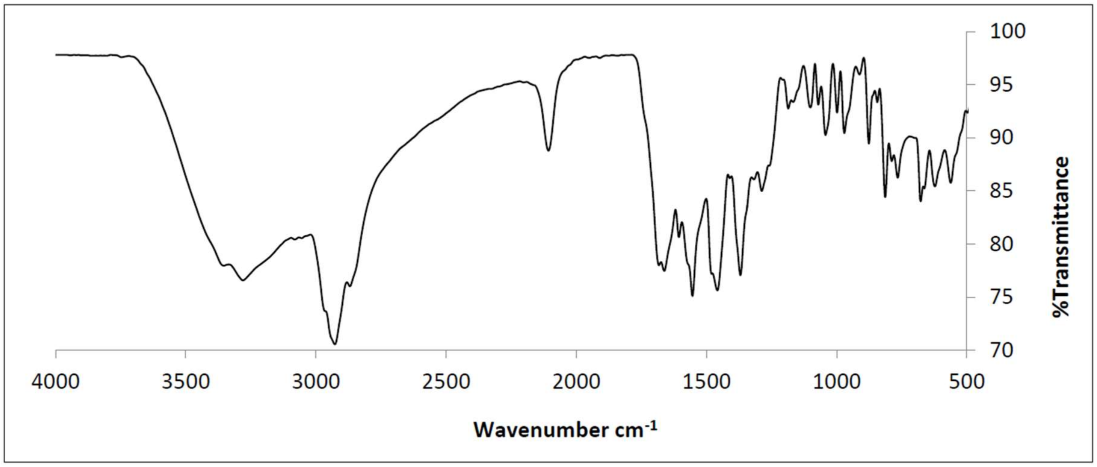

Figure S15: Compound **16**

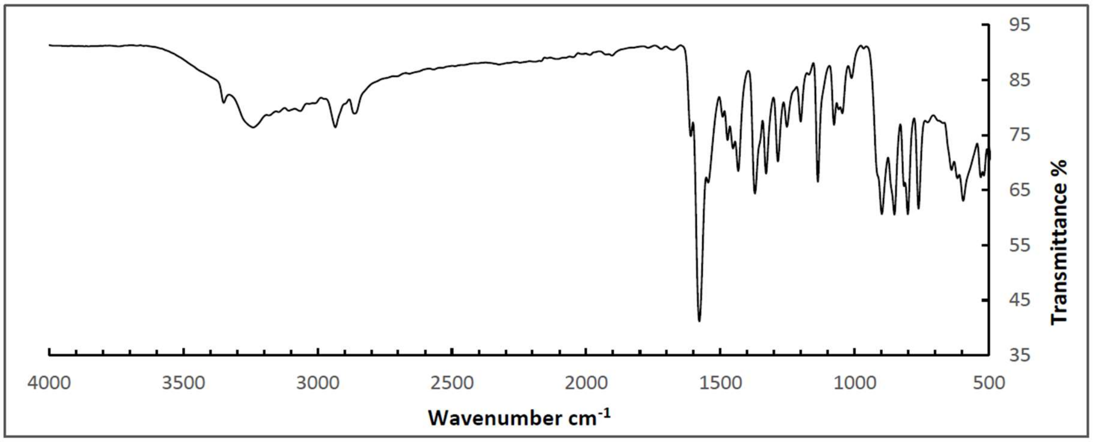

Figure S16: Compound **17**

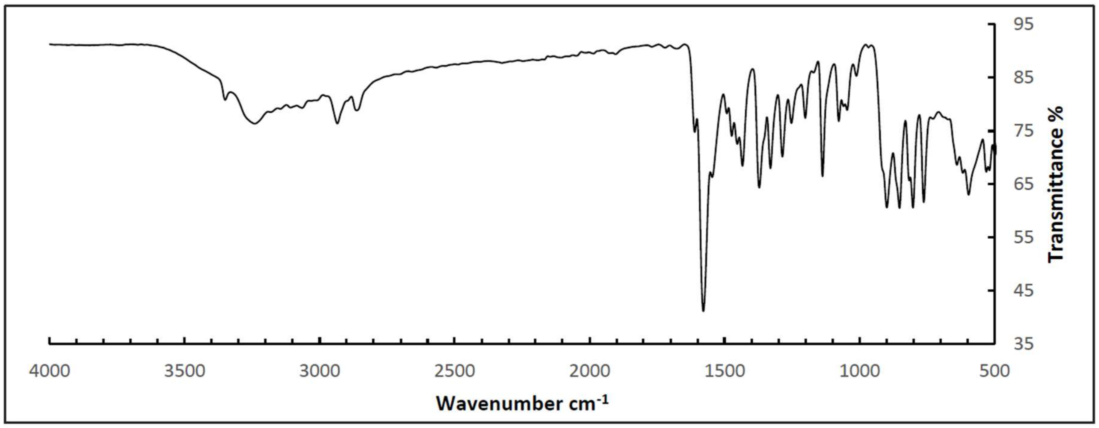

Figure S17: Compound 18

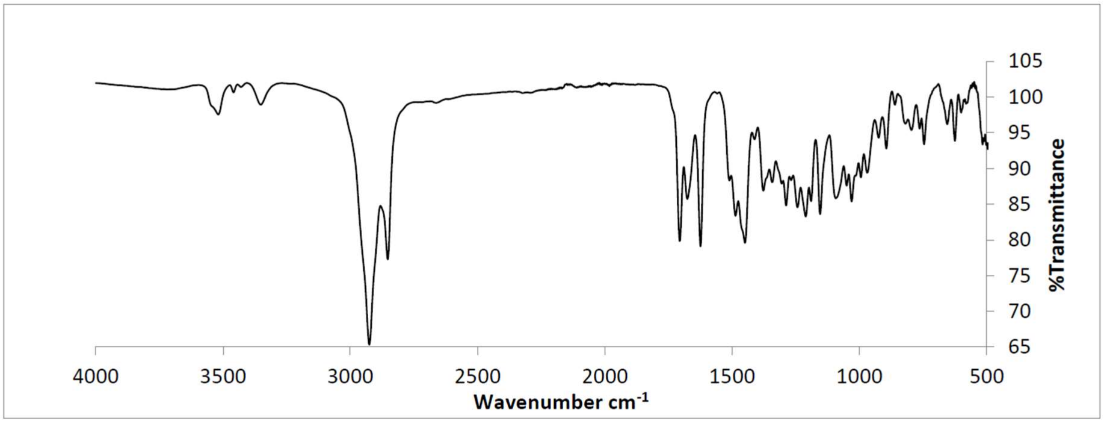

Figure S18: Compound **19**

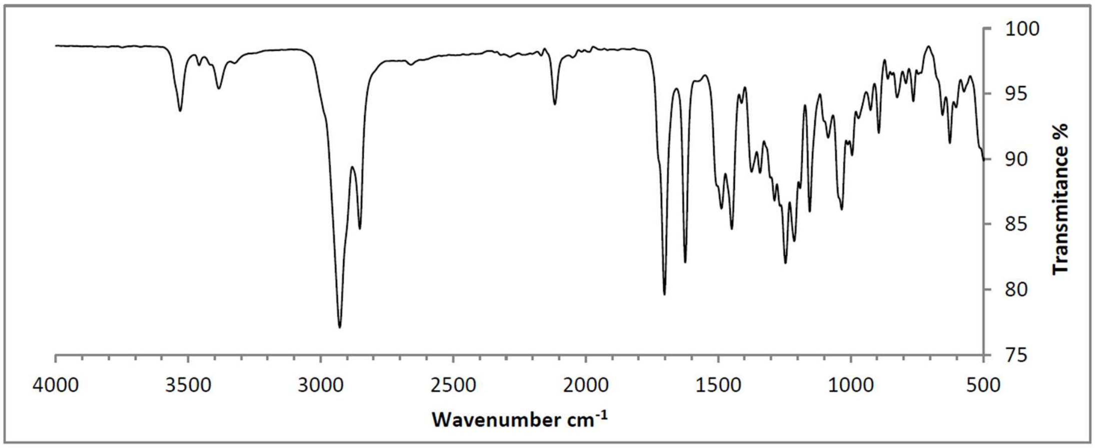

Figure S19: Compound **21**

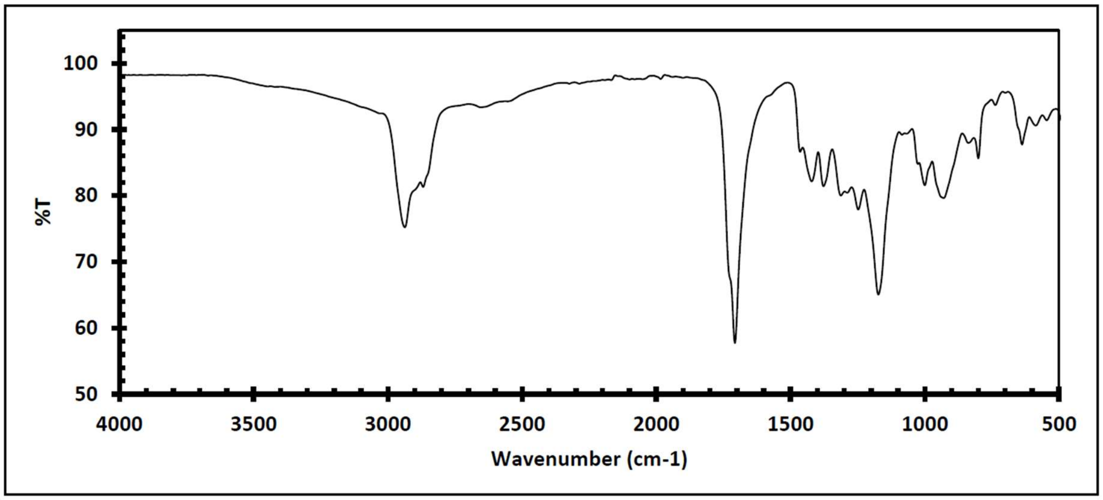

Figure S20: Compound **23**

LCMS spectra of compounds **12–19**, **21-23** (Figure S**11**-Figure S**20**)

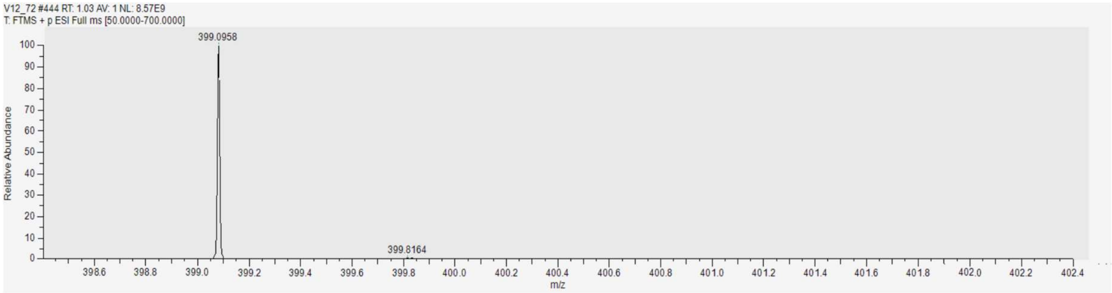

Figure S21: Compound **12**

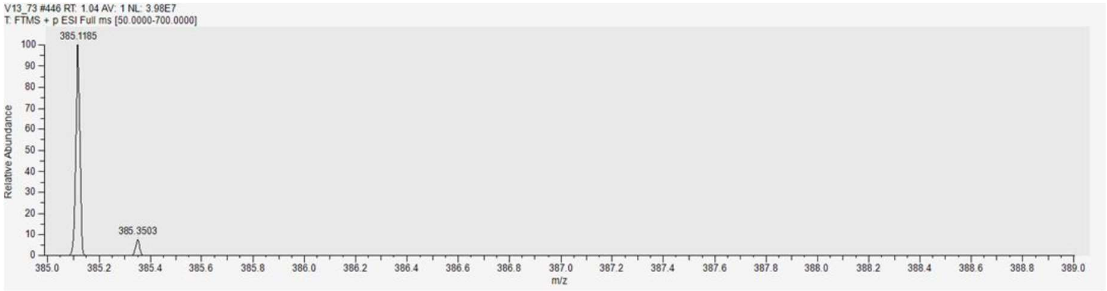

Figure S22: Compound **13**

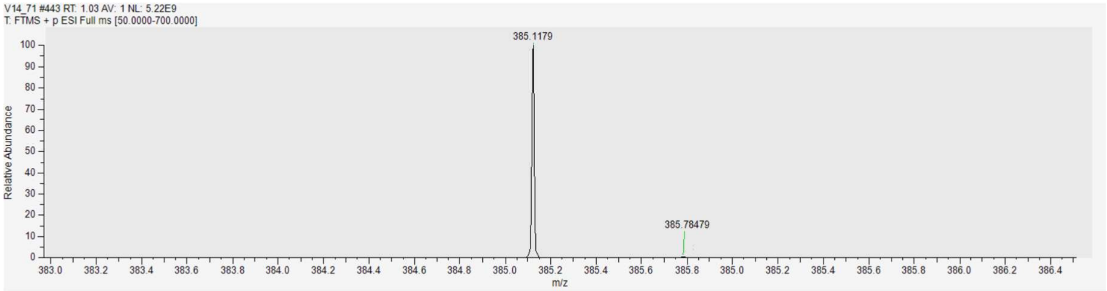

Figure S23: Compound **14**

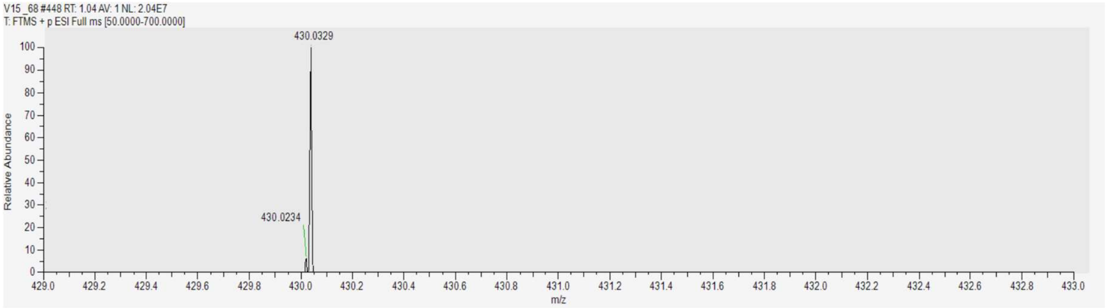

Figure S24: Compound **15**

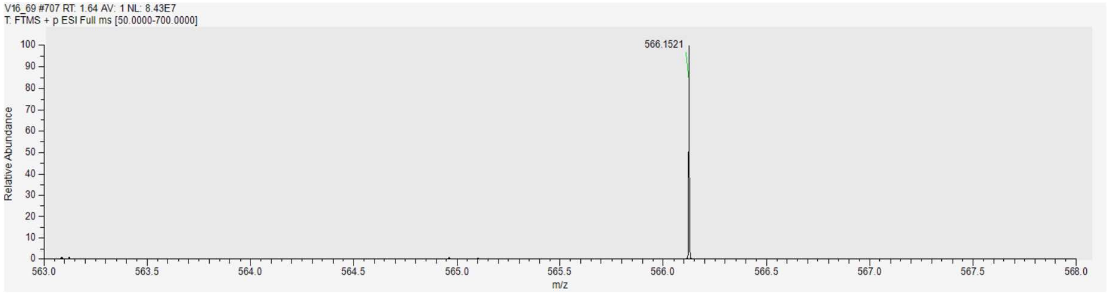

Figure S25: Compound **16**

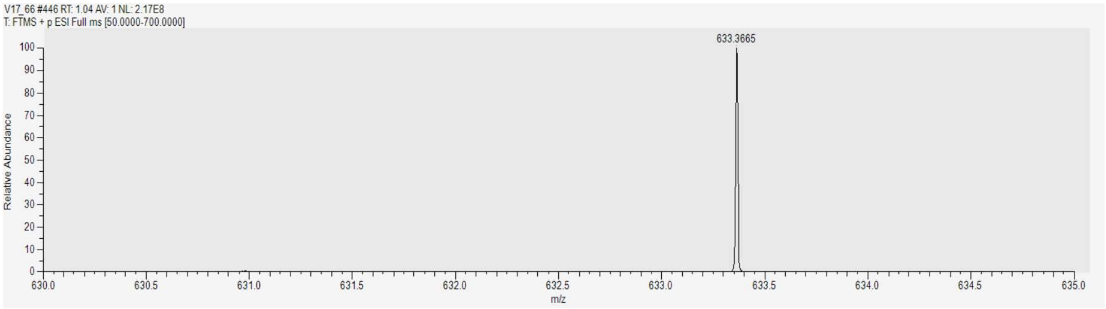

Figure S26: Compound **17**

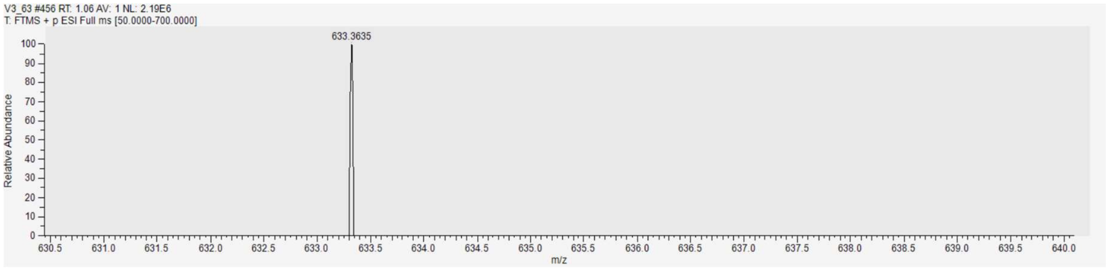

Figure S27: Compound **18**

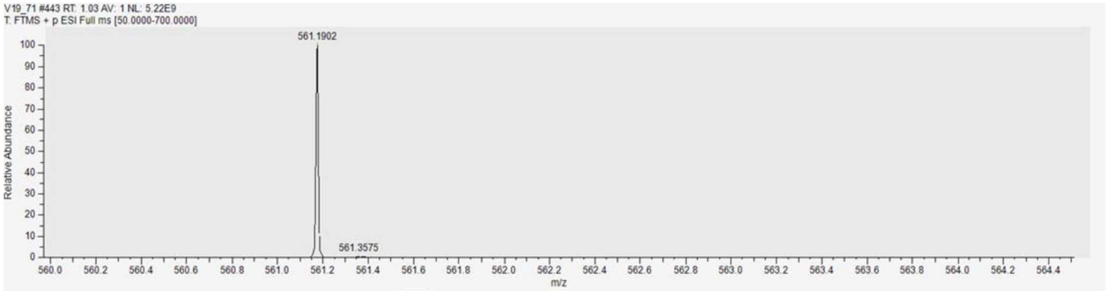

Figure S28: Compound **19**

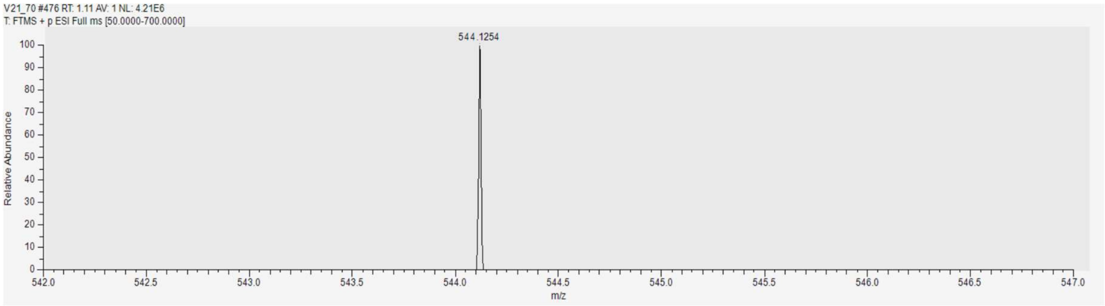

Figure S29: Compound **21**

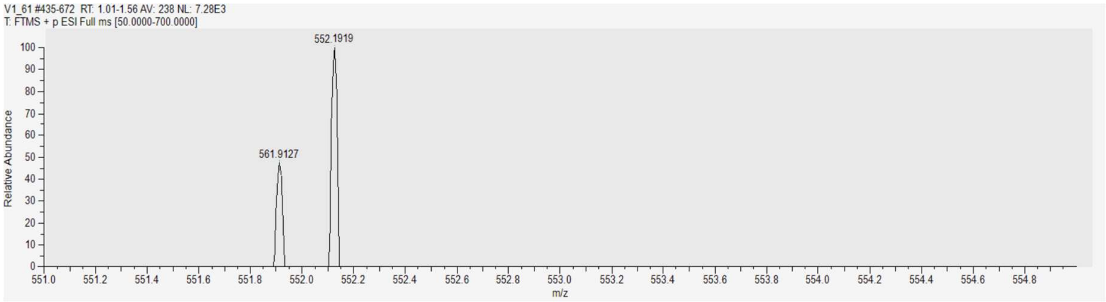

Figure S30: Compound **23**
